# Thigh-Derived Inertial Sensor Metrics to Assess the Sit-to-Stand and Stand-to-Sit Transitions in the Timed Up and Go (TUG) Task for Quantifying Mobility Impairment in Multiple Sclerosis

**DOI:** 10.3389/fneur.2018.00684

**Published:** 2018-09-14

**Authors:** Harry J. Witchel, Cäcilia Oberndorfer, Robert Needham, Aoife Healy, Carina E. I. Westling, Joseph H. Guppy, Jake Bush, Jens Barth, Chantal Herberz, Daniel Roggen, Björn M. Eskofier, Waqar Rashid, Nachiappan Chockalingam, Jochen Klucken

**Affiliations:** ^1^Brighton and Sussex Medical School, University of Sussex, Brighton, United Kingdom; ^2^Friedrich-Alexander-Universität Erlangen-Nürnberg, Erlangen, Germany; ^3^Centre for Biomechanics and Rehabilitation Technologies, Staffordshire University, Stoke-on-Trent, United Kingdom; ^4^School of Media, Film and Music, University of Sussex, Brighton, United Kingdom; ^5^ASTRUM IT, GmbH, Erlangen, Germany; ^6^Department of Engineering and Design, University of Sussex, Brighton, United Kingdom; ^7^Hurstwood Park Neuroscience Centre, Haywards Heath, United Kingdom; ^8^Molekulare Neurologie, Universitätsklinikum Erlangen, Erlangen, Germany

**Keywords:** wearable, gyroscope, gait, mobility, walking, standing, sitting, accelerometer

## Abstract

**Introduction:** Inertial sensors generate objective and sensitive metrics of movement disability that may indicate fall risk in many clinical conditions including multiple sclerosis (MS). The Timed-Up-And-Go (TUG) task is used to assess patient mobility because it incorporates clinically-relevant submovements during standing. Most sensor-based TUG research has focused on the placement of sensors at the spine, hip or ankles; an examination of thigh activity in TUG in multiple sclerosis is wanting.

**Methods:** We used validated sensors (x-IMU by x-io) to derive transparent metrics for the sit-to-stand (SI-ST) transition and the stand-to-sit (ST-SI) transition of TUG, and compared effect sizes for metrics from inertial sensors on the thighs to effect sizes for metrics from a sensor placed at the L3 level of the lumbar spine. Twenty-three healthy volunteers were compared to 17 ambulatory persons with MS (PwMS, HAI ≤ 2).

**Results:** During the SI-ST transition, the metric with the largest effect size comparing healthy volunteers to PwMS was the Area Under the Curve of the thigh angular velocity in the pitch direction–representing both thigh and knee extension; the peak of the spine pitch angular velocity during SI-ST also had a large effect size, as did some temporal measures of duration of SI-ST, although less so. During the ST-SI transition the metric with the largest effect size in PwMS was the peak of the spine angular velocity curve in the roll direction. A regression was performed.

**Discussion:** We propose for PwMS that the diminished peak angular velocity during SI-ST directly represents extensor weakness, while the increased roll during ST-SI represents diminished postural control.

**Conclusions:** During the SI-ST transition of TUG, angular velocities can discriminate between healthy volunteers and ambulatory PwMS better than temporal features. Sensor placement on the thighs provides additional discrimination compared to sensor placement at the lumbar spine.

## Introduction

Multiple Sclerosis (MS) is a progressive neurological disorder usually presenting in early adulthood whose manifestations include an unpredictable spectrum of motor, sensory and autonomic symptoms, usually accompanied by increasing levels of ambulatory dysfunction (1, 2). The relapsing-remitting form of the disease (RRMS) involves attacks of sudden exacerbations of symptoms lasting days to weeks, caused by autoimmunity, inflammation and demyelination, followed by abatement of many (but not all) of the new symptoms during periods of remission. Although MS is currently without a cure or a known cause, the last decade has seen a renaissance in disease modifying treatments and symptomatic therapies (3). Researchers' goals are to find new medical and physiotherapy treatments that can improve function after an attack and prevent new attacks (4), greatly improving the quality of life of patients. Assessment of intervention efficacy fundamentally depends on making accurate measurements of disease progression and disability.

### Traditional measurements of disability progression in MS

Objective and precise measurements of movement disability (including weakness and attenuation of coordination and control) are needed to make clear assessments about interventional efficacy and disease symptom progression (5). However, the day-to-day variation in MS symptom severity, combined with the relapsing-remitting course of RRMS, undermine precise assessment of symptomatic progression at a given moment in time. Furthermore, the efficacy of new treatments is sometimes disputed because of issues associated with the disability outcome measures (6, 7). Current interventions (including medications and physiotherapy) used to treat MS symptoms are often modestly effective, and may exert their clinical effects on only a small subpopulation of those treated. For example, fampridine (4-AP) was shown to elicit a 25% improvement in ambulation of MS patients (compared to 6% in placebo-treated patients), but only in 35% of such patients (8).

There is a correlation between clinical progression, as implied by MRI measures of brain atrophy and gross tissue loss, and symptomatic progression, although more fine-grained MRI measures of disease activity such as T2 lesion load do not always correlate directly with overall symptomatic assessment such as with the Multiple Sclerosis Functional Composite Score (MSFC) (9) or with validated tools based on clinical judgment such as the EDSS (Expanded Disability Status Scale) (10). In summary, both research and treatment into MS are characterized by uncertainty because it can be difficult to quantify modest improvements due to treatments (11, 12).

### Inertial sensors and other metrics of mobility dysfunction

In general, detailed measurements of gait function and mobility require a specialist gait laboratory setting (e.g., for opto-electronic motion capture) and are too costly, isolated and time-consuming for routine clinical use. Inertial Motion Units (IMUs) are a cost-effective, wearable subclass of wireless sensors based on Micro-Electromechanical Sensor (MEMS) technology, which often include a collection of accelerometers, gyroscopes and magnetometers, allowing the derivation of motion of various body segments; the choice of which body segment (e.g., ankle, hip, thigh, or a combination) will provide the minimal sensitivity needed to interpret the task remains controversial (13). Recent research has highlighted the opportunities for use of inertial sensors in MS (14), although most of this work has focused on home-based measures of total physical activity (15), with a comparatively smaller number of attempts to characterize walking in MS (16, 17). By contrast, in other causes of movement disorder [e.g., Parkinson's disease (18), stroke (19), total knee arthroplasty (20), and elderly patients at risk of falling (21)], there is a broader range of data considering the strengths and weaknesses of the sensor metrics. Recently, at the level of the thigh, hip range of motion (ROM) has been found to be a useful metric to assess disability in MS during flat walking (17). In addition to walking, sensor measurements of ambulatory ability are broadened by a wide range of clinically-established tasks that the patient can perform.

### TUG and other tasks

In the International Classification of Functioning [ICF (22)], the domain of activities can be broken down into capacity and performance. While direct tests of muscle strength arising during maximal isometric contraction can be measured with a force transducer, to assess clinically relevant disability, muscle actions are usually assessed within a more naturalistic context, such as walking a short distance, walking a longer distance (where fatigue and walking degradation are possible), or getting out of a chair and starting to walk. The Timed-Up-And-Go (TUG) task (23) tests the time it takes for a patient to stand up from a seated position, walk 3 m, turn around 180°, walk back 3 m, turn around and sit back down again; the task begins when the clinician gives the signal to start, and it ends when the patient's body first returns to the seat pan of the chair. TUG duration is a modest predictor of frailty and falls (24), and TUG is a threshold test for independent living. In their original, non-instrumented format, most of these naturalistic tasks had only a single metric output, which was either time duration (e.g., TUG) or distance covered successfully (e.g., the 6 min walk).

**Figure 1:**
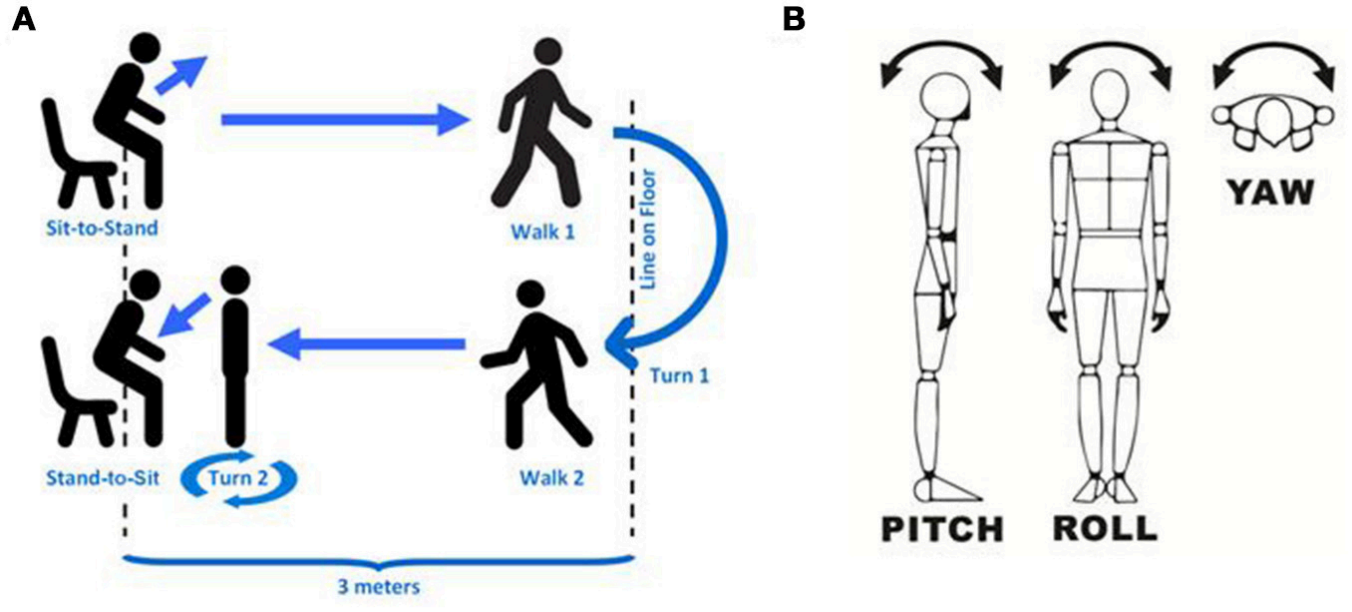
Clarification of methods. **(A)** shows a schematic of the entire TUG task divided into subtasks. **(B)** shows the approximate directions of pitch, roll and yaw (depending on precise sensor stability) as we describe in this study. Pitch is nominally rotation around the medio-lateral axis (i.e., within the sagittal plane), roll is nominally rotation around the dorso-ventral axis (i.e., within the coronal plane), and yaw is nominally rotation around the vertical (superior-inferior) axis (i.e., within the transverse plane).

TUG can be effectively considered as six subtasks (Figure 1A): the sit-to-stand transition (SI-ST), walking 1 (away), turn 1 (180°), walking 2 (return journey), turn 2 (180°), and the stand-to-sit transition (ST-SI); in analyses, walking 1 and walking 2 are often bundled together because they represent nearly identical subtasks, and some analyses elide turn 2 with the ST-SI transition because the two subtasks usually do not have a clear boundary. A range of TUG-like variants also exist that shorten the walk [8-UG (25)] or lengthen it [to 7 m each way (26)] in order simplify the task for patients or to make the walking data more robust.

### The sit-to-stand transition and the thigh

What makes TUG and TUG-like tasks different from other walking tasks (e.g., the Timed-25 Foot Walk or the 6 min Walk) is the inclusion of the sit-to-stand transition and the stand-to-sit transition [also some researchers have also investigated aspects of the turns (16, 27)]. The sit-to-stand transition (and the continuation into walking) is not only ecologically relevant for day-to-day living, but it is particularly affected in the frail elderly who complain of stiffness after extended sitting. It is also highly dependent on extensor strength in the lower extremity, and is considered one of the most mechanically demanding of functional daily activities (28). The stand-to-sit transition is an indicator of control and balance during eccentric contraction of the extensors. For stroke, specific SI-ST metrics (such as rising speed or asymmetry of weight distribution) have been proposed as possible metrics for detecting improvement during the first year post-stroke (29, 30). The asymmetry features are particularly important in stroke because of hemiparesis, although rising speed might potentially be useful in any movement disorder, including MS; to the best of our knowledge a similar investigation for MS has not occurred.

Some groups have looked at single SI-ST transitions, or cycles of Sit-Stand-Sit transitions, which provide more uniform data about the SI-ST transition, because TUG often results in elision of the SI-ST transition and walking 1 when the first step (toe-off and swing) begins before or immediately at the completion of contralateral thigh extension. Compared to the ankles, the SI-ST transition has a profound effect on the directionality of the thigh segment (and to the torso as well).

### Known sensor metrics for TUG

Extensive sensor-based research on TUG has been performed in a range of clinical conditions (31, 32). A brief survey of this literature reveals at least 90 sensor metrics for TUG have been derived to recognize falling risk. In a 2014 systematic review of 53 sensor-based studies on the sit-to-stand transition (32), 84% of the studies used a sensor on the torso, at either the spine [e.g., L3 (33, 34)] or the sternum [e.g., (18)]. Other studies have placed sensors on the shanks (16, 27, 35); only in a few cases placement was on the thigh segment (20, 36, 37), despite the fact that the thigh would be the most directly involved body segment during the SI-ST or ST-SI transition. The many metrics (based on all body segments) have included calculations based on temporal variables, linear acceleration variables, angular velocity variables, frequency variables, and descriptive statistics based on entropy (ApEn) and fractal dimension (d_F_). Some groups have measured asymmetry in weight bearing (36). The derived temporal variables (and asymmetry) are the most clearly related to traditional gait measures (which are based on position and force), while sensor metrics are based on movement (angular velocity and linear acceleration).

In the current study we sought to compare a collection of transparent metrics of the SI-ST and ST-SI transitions, assessing whether there was added value when measurements were made with sensor placement at the thigh, compared to placement at the spine. We judged assessment value in terms of effect size (the rank biserial) of the association of a feature with its ability to distinguish middle-aged healthy participants from Persons with MS (PwMS). In addition to temporal measures, we examined a range of calibrated, transparent sensor metrics, as well as testing two different measures of the smoothness of signals. As a rough test of whether our metrics would be useful in examination of PwMS, we compared an ambulatory sample of MS patients [Hauser Ambulation Index (HAI) ≤ 2, no use of walking aids for short distances] to middle-aged healthy volunteers. Thus, our hypothesis is that there exists a set of thigh-based sensor metrics of pitch angular velocity that have a higher effect size in distinguishing PwMS from healthy volunteers than either the TUG stopwatch time or the published spine based metrics. Finally, to roughly simulate the value of our features, we produced a step-wise logistic regression with multiple features.

## Methods

### Volunteer recruitment

Seventeen PwMS (mean age ± sd = 53.06 ± 11.06, 13 female) were recruited from a local community MS center (MS Sussex), with approval from Staffordshire University ethics committee. Twenty-three healthy volunteers (age 46.13 ± 11.12, 14 female) were recruited from the university community via email. The exclusion criteria were that no participant had clinically relevant complicating diseases (other than MS) that would impact walking ability or walking rates. This included: not currently suffering from flu, cold, etc., no current leg/back injuries due to trauma, no loss of motivation due to obvious psychiatric symptoms (e.g., no major depression, bipolar disorder, psychosis), and no loss of walking ability or exercise tolerance due to another disorder: heart failure, recent myocardial infarction, COPD or other respiratory disorder.

### Procedure

The experimental procedure was approved by the university ethics committee, and the experiment was run according to the principles in the Declaration of Helsinki. Each participant was informed about the nature of the experiment, and they gave their informed consent for the experiment. Before each volunteer began, he/she filled in a demographic form (establishing their age and gender, estimated year of first symptoms, and year of receiving an MS diagnosis). Three of our sensors were non-invasively placed on the lateral aspect of their lower left thigh (the most distal part of the sensor was 5 cm above the superior border of the patella), lower right thigh, and the small of the back (at the level of L3). All sensors were worn over clothing using a lightweight Velcro elasticated webbing system for keeping the sensors in place. All participants wore standardized running shoes (Lonsdale) of the correct shoe size, in order to correct for differences in mobility due to shoe stiffness or heels; our team have a collection of different sizes of these running shoes to fit all participants. Sensors were placed on the lateral surfaces of thighs, to avoid interference with walking; sensors were orientated with the positive X-axis pointing superiorly (proximally).

### Patient reported outcome measures

The participant filled in six forms: the self-assessed version of the Extended Disability Status Scale [EDSS-S, (38)], the Multiple Sclerosis Walking Scale [MSWS-12 (39)], the Fatigue Severity Scale [FSS, (40)], the Modified Fatigue Impact Scale [MFIS, (41)], the Beck Depression Inventory [BDI, (42)], the International Physical Activity Questionnaire (IPAQ-short) (43). These six scales (plus the demographics scale) required approximately 20 min to fill in.

Fitting the sensors took 5 min, while removing the sensors took 3 min. In general the entire procedure for a single volunteer lasted 60 min (including rest time). The sensors had their data synchronized at the beginning and the end of the experiment by being affixed together and being subjected to sudden transient accelerations, interspersed with periods of non-movement.

### Tasks

The timed-up-and-go (TUG) task was performed according to Steffen et al. (44). The task involves arising from a seated position, walking 3 m, turning around, walking back 3 m, turning around and sitting back down in the chair. Participants started in a chair with arms, with a tape mark on the floor showing the 3 m distance where they were supposed to turn around. Participants were given instructions to perform the task “as fast as possible, but safely,” and they were shown how to do the task. Stopwatch timing was done according to best practice (44, 45), starting on the word “Go” and ending when the participant's buttocks first made contact with the seat of the chair; a sensor-based full length TUG duration feature was also calculated based on the attitude of the thigh. The TUG task was performed twice.

Participants were also asked to perform several other walking and balance tasks, including a Timed-25-Foot-Walk [T25FW based on timing with a stopwatch, (46)], which was used to establish that participants were at the Hauser Ambulation Index [HAI, (47)] of 2 or below. None of the tasks were stressful or tiring, and participants were asked before each task if they needed a rest.

### Sensors and data analysis

The sensors used were x-IMU by X-io (Bristol, UK), with three dimensions each of accelerometry, gyroscopy and magnetometry. These sensors are factory calibrated for gravitational acceleration (accelerometers) and angular momentum (gyroscopes), and they incorporate an onboard algorithm for estimation of heading and quaternions (48, 49). These sensors have been validated for accuracy when measuring walking, both in terms of angular velocities and derived temporal gait metrics (50). Data from the three sensors in each x-IMU node was gathered at 128 Hz onto the onboard 32 GB micro SD cards (Sandisk Ultra Micro) with the sensors' blue tooth transmission off (to extend battery charge). Time alignments between sensors and with other measurements and video tapes were performed using an automated event-based synchronization strategy [e.g., (51)]. Directions used (i.e., pitch, roll and yaw) are shown in Figure 1B.

Binary file sensor data was transferred to a Windows 7 computer, and the binary files were converted into csv files using the manufacturer's provided Graphical User Interface. The csv files were read into Matlab, and all sensor data was aligned (based on the synchronization signals at the beginning and end of the experiment) with a purposed-made script; timing differences between sensors were interpolated linearly–at no point did the original sensor acquisition data differ between sensors by more than 50 ms (over the course of 90 min of acquisition).

**Figure 2:**
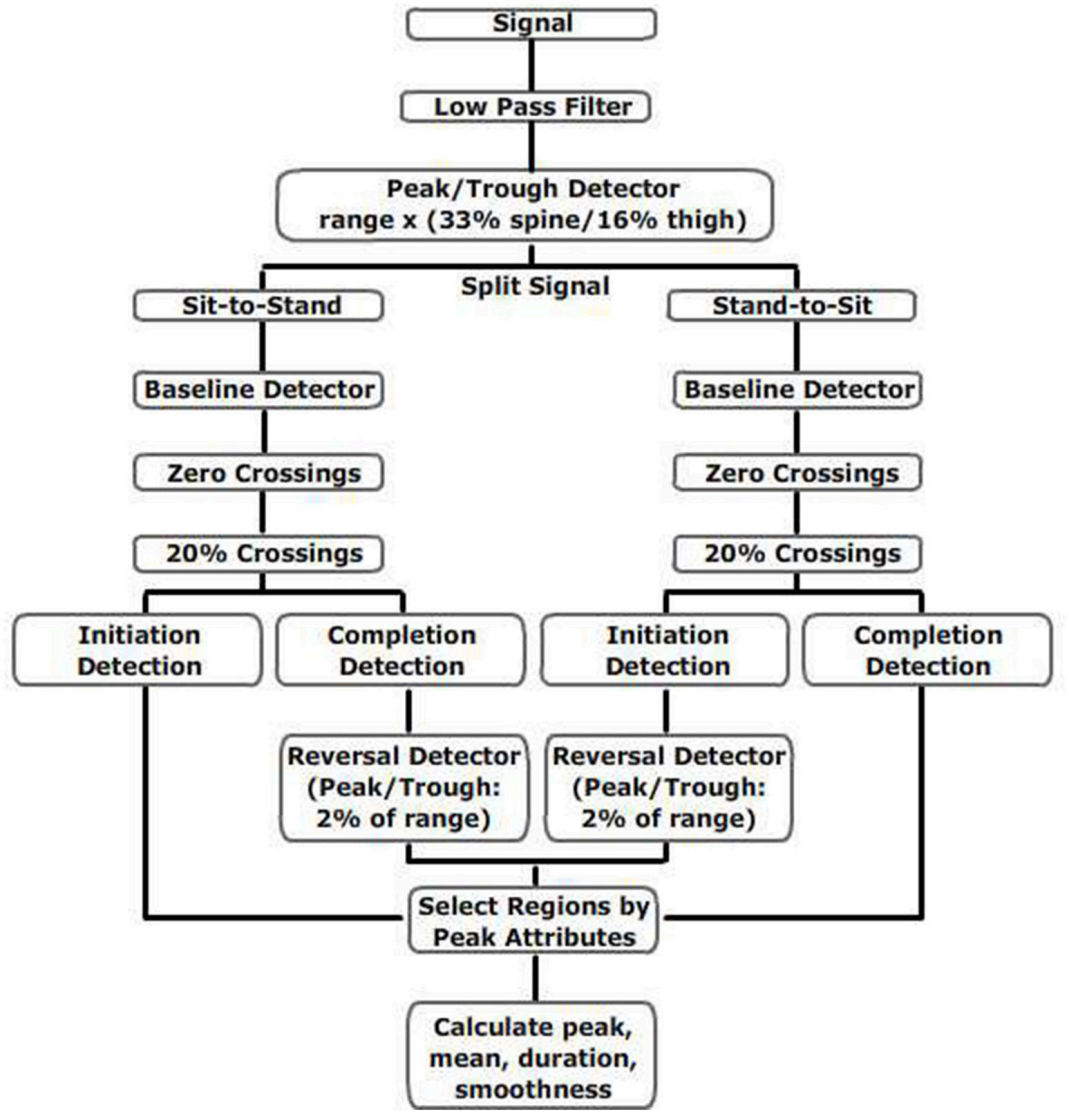
Flow chart for calculations of thigh and spine measures during TUG.

The relevant sensor data for each task was located by Matlab based on the event's start and finish time recorded by the sensor, and all data was low-pass filtered (2.5 Hz, 4th order Butterworth, 0 latency, Matlab filtfilt). Peaks were identified with a peak detector algorithm set to detect a minimum recovery of 20% of the range of the signal. Timing duration from the spine sensor was based on Weiss et al. (33, 52), while all other angular velocity and duration measurements were derived as shown in Figure 2.

### Smoothness

To test control of movement, repeated gait movements can be tested for variation, such as the Coefficient of Variation for any metric (e.g., step length) (53). For a single movement performed once (e.g., the SI-ST transition), inconsistent neural control (or loss of balance) may be reflected by a loss of smoothness (which is often measured as an increase in jerk for a continuous signal). In this study, we tested two different measures of smoothness. The normalized mean absolute jerk (54) is one of the most commonly used measures for smoothness (smoothness 1):

$$\begin{array}{l} \eta_{\text{nmaJ}}≜-\frac{1}{\upsilon_{peak}\left(t_{2}-t_{1}\right)}\overset{t_{2}}{\underset{t_{1}}{\int }}|\frac{d^{2}\upsilon }{dt^{2}}|dt \end{array}$$

Another measure of smoothness we used, the speed arc length (55), has the advantage of being unit-free (smoothness 2):

$$\begin{array}{l} \eta_{spal}\overset{\Delta }{=}\;-\text{ln}\left(\overset{t_{2}}{\underset{t_{1}}{\int^{\text{​}}}}\sqrt{{\left(\frac{1}{t_{2}-t_{1}}\right)}^{2}+{\left(\frac{d\hat{\upsilon }}{dt}\right)}^{2}}dt\right)\\ \;\hat{\upsilon }(t)\overset{\Delta }{=}\frac{\upsilon (t)}{\upsilon_{\text{peak}}}. \end{array}$$

### Statistics

Statistics were calculated within Matlab (Natick, MA, USA). To allow for peaks from different legs (and in different directions) to be compared, all peaks are the peak of the absolute value of the calibrated signal, and all means are also the mean of the absolute value of the calibrated signal.

Graphical inspection of healthy and PwMS peak angular velocity data showed that it was approximately normally distributed; nevertheless, to allow for those features that were not normally distributed, for assessments of correlation between repeated attempts of the same task, an Intraclass Correlation Coefficient (ICC) was calculated (56). For unpaired comparisons between the means of two populations, the Wilcoxon Rank Sum test was used; this was corrected by the Holm-Bonferroni correction for multiple comparisons. For effect size calculations, the rank biserial was calculated.

## Results

### Participants

The two cohorts compared in the main study were ambulatory persons with multiple sclerosis (PwMS) and middle-aged healthy volunteers. The PwMS were recruited via a local MS community center (MS Sussex Treatment Center). The baseline characteristics of the two groups are shown in Table 1. The two groups were not statistically significantly different in terms of height, weight, or age (although the mean age difference was >6 years). In all other measurements of disability and difficulty, the PwMS had significantly higher Beck Depression Index Scores, MSWS-12 scores, FSS scores, MFIS scores, and T25FW times (which were on average 1.5 s longer than the times for healthy volunteers). This difference in mean T25FW is just over the established cut-off of 20% that suggests a clinically meaningful difference (46), and the mean of 6.02 s is almost exactly the 6 s cut-off established for clinically meaningful cut-off (57).

**Table 1:** Baseline characteristics of participants.

**Variable**	**PwMS**		**Healthy**		
*n*	17		23		
Gender (f/m)	13/4		14/9		
IPAQ (high/medium/low)	3/9/5		11/10/2		
**Variable**	**Mean**	**St. Dev**.	**Mean**	**St. Dev**.	***P***
Age (years)	53.06	±11.06	46.13	±11.12	NS
Height (cm)	167.8	±11.2	170.1	±10.4	NS
Weight (kg)	74.9	±26.2	70.6	±11.2	NS
EDSS-S	4.00	±1.80	0.1	±0.2	< 0.0001
Beck depression index	11.8	±8.2	5.6	±9.8	< 0.001
MS walking scale-12	50.6	±21.5	0	±0	< 0.0001
Fatigue severity scale	5.0	±1.5	2.8	±1.3	< 0.001
Mod. fatigue impact Sc	42.2	±21.5	15.7	±17.0	< 0.001
Timed 25 foot walk (s)	6.02	±1.23	4.53	±0.68	< 0.0001
Timed up-and-go (s)	12.44	±2.70	10.27	±1.53	< 0.05

*P-values are based on the Wilcoxon Rank Sum Test. PwMS, Persons with Multiple Sclerosis; n, total number of participants in that category; St. Dev., standard deviation; NS, not significant. f/m, female/male; IPAQ, International Physical Activity Questionnaire; Mod Fatigue Impact Sc, Modified Fatigue Impact Scale (MFIS); EDSS-S, Self-Administered Expanded Disability Status Scale*.

### Format of TUG data

**Figure 3:**
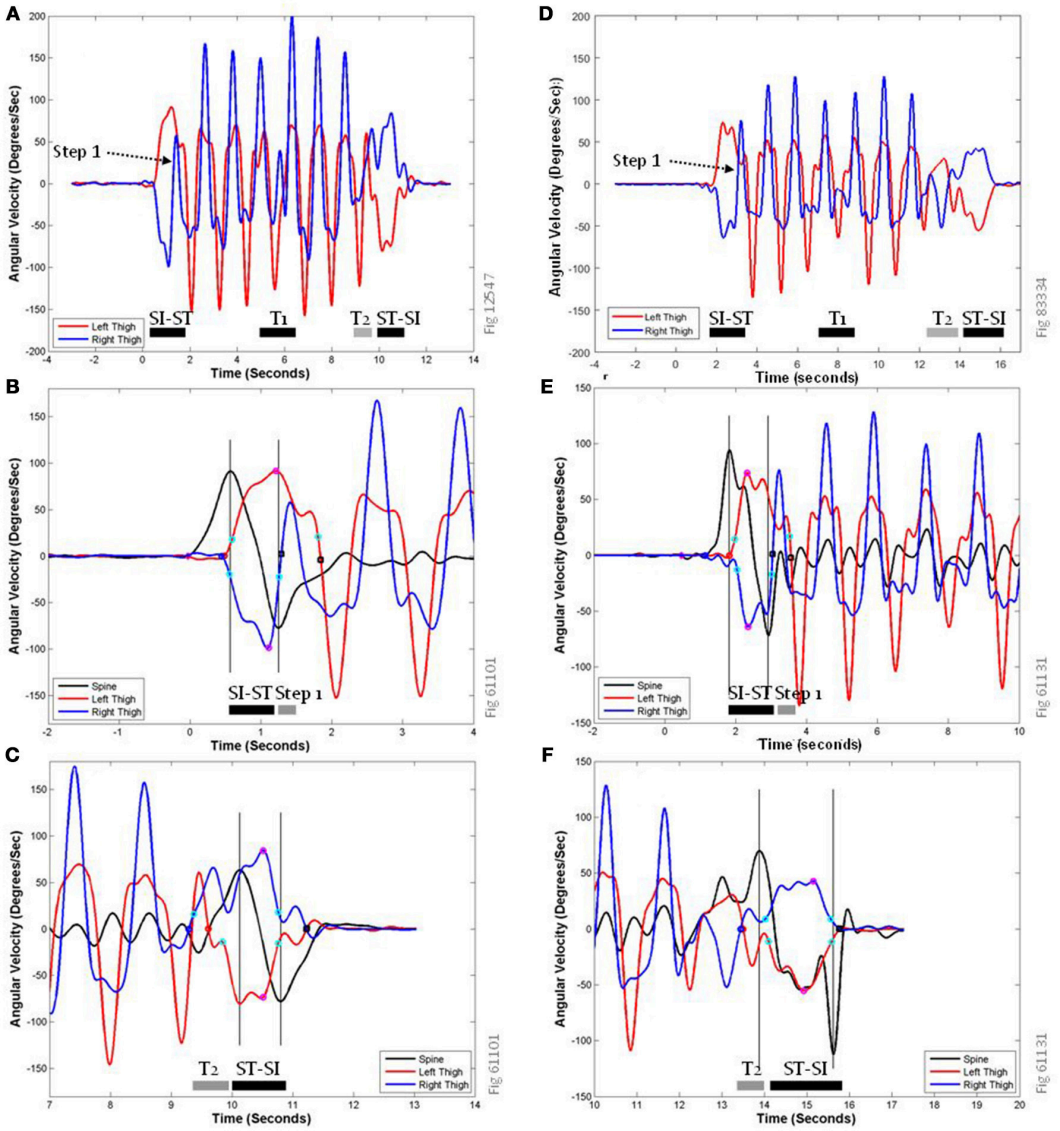
Representative traces of pitch gyroscope sensors data during the TUG task. **(A)** shows the activity of the left (red) and right (dark blue) thigh sensors during the entire TUG task for a healthy volunteer. SI-ST, sit-to-stand transition; ST-SI, Stand-to-Sit transition; T1, Turn 1; T2, Turn 2; which elides directly into the ST-SI transition. **(B,C)** show expanded views of the same representative traces at the sit-to-stand transition **(B)** and the stand-to-sit transition **(C)**, labeled with key points for feature calculation. **(D–F)** show analogous traces for a PwMS; note that the different panels have slightly different scales on their axes. In addition to the pitch traces from the left thigh (red) and the right thigh (dark blue), **(B,C,E,F)** include a pitch trace from the lumbar spine sensor (black), to allow comparisons with previously published data features based on torso-mounted sensor data. The peaks/troughs for the thigh traces are magenta circles, and the peaks/troughs for the spine are shown as vertical gray lines. The start of the rise for the left thigh is a red circle, for the right thigh is a dark blue circle, and for the spine is a magenta diamond. Step end points are shown as black squares, and 20% rise and 80% return points are shown as cyan circles.

Pitch gyroscope data from each sensor (and roll data from the lumbar spine sensor) were used to derive both the rate of movement during the sit-to-stand (and stand-to-sit) transitions, as well as the durations that these activities lasted. The features we calculated were based on finding peaks, calculating the peak attributes (maximum, start point, end point, 20% rise point, 80% return point), and from those points calculating the magnitude of the peak (angular velocity), the duration (time in seconds) of the peak's arc (where an arc is the geometric segment of the angular velocity curve), the mean angular velocity of the peak's arc, the area under the curve of the arc, and the smoothness of the peak's arc. Representative sensor data is shown in Figure 3. All traces in this figure are low pass filtered (2.5 Hz) and factory-calibrated. Sharp peaks/troughs correspond to the thigh's role in swing phase, while wider simultaneous peaks/troughs are the stance phase of the contra-lateral lower limb. Panels A (healthy) and D (PwMS) show both left and right thigh pitch traces during the entire TUG task; each walking step is clearly identifiable from the swing phase (sharp peaks) and concurrent contra-lateral stance phase (wider, blunt peaks), as are the sit-to-stand and stand-to-sit transitions (wider and lower-amplitude changes). The turns are more easily identified by the traces for the yaw gyroscopes (not shown). The first half step (“step 1”) that occurs immediately after standing up entails a small swing phase (in panel A it is the right thigh trace between 1.2 and 1.5 s) that peaks at a much lower angular velocity than other steps.

Figure 3B is a close up of panel A during the sit-to-stand transition showing the relationship between the peaks of the spine pitch trace (black line) and the thigh traces. In previous studies (33, 52), the spine pitch trace was the data used to derive the timing of the SI-ST and ST-SI transitions. For this volunteer, the first spine peak (intersection of black time course trace and left-most vertical gray line) is closely aligned with the initiation of thigh movements (red and dark blue circles), and the second spine peak/trough (right-most vertical gray line) is closely aligned with the beginning of the first half step (i.e., one possible end of the sit-to-stand transition). For the purposes of computer identification, zero-crossing points of the thigh traces (black squares) were used as markers for the end of SI-ST transitions. Panel C is a close up of panel A during the stand-to-sit transition showing the relationship between the peaks of the spine pitch trace and the thigh traces; for this volunteer, the second spine peak (right-most vertical gray line) is closely aligned with the thighs' return to the seat pan of the chair (i.e., the end of the stand-to-sit transition), which is identified by the 80% return point (cyan circles). The delay of the thigh pitch traces (red and blue traces, between 10.8 and 11.3 s) to arrive at 0°/s (black squares) in this case is due to abduction/adduction of the thighs accompanied by thigh rotation, rather than a delay in sitting (i.e., the hands bracing against the fall downward). The first spine peak is delayed compared to knee and thigh flexion (cyan circle on red line at 9.8 s). The thigh activity of the right lower limb (dark blue) is a combination of the final shuffling step during Turn 2 (T2, starting at the dark blue circle) and the subsequent flexion of sitting down.

The traces related to a PwMS in panel D show a similar set of activities as in panel A, although the actions are performed more slowly and with lower angular velocity peaks. The most noticeable difference is that in panel F the ST-SI transition is performed much more slowly and carefully.

**Figure 4:**
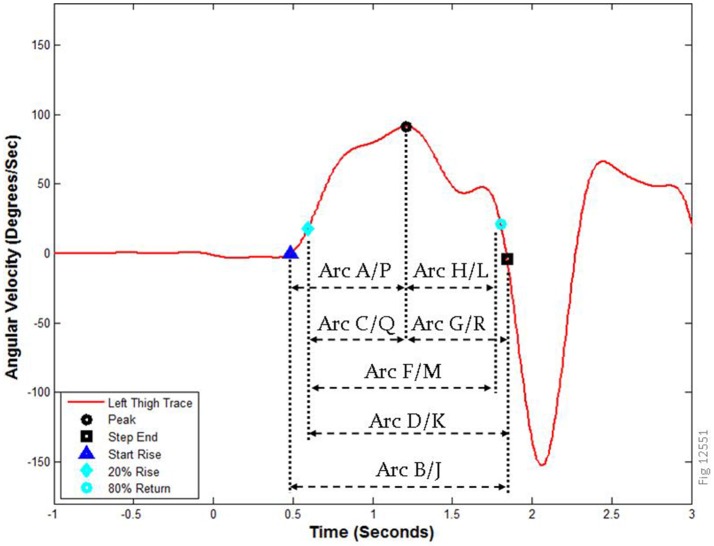
Arc boundaries used for calculations of features. The left thigh pitch trace from the sit-to-stand transition in Figure 3 is labeled with the relevant time markers and peak attributes used to calculate the features in this study. Arcs A–H correspond to the SI-ST transition, while arcs J–R represent the ST-SI transition. How these points were computationally derived is described in the methods; note that arcs E and N (not shown) are 1 s regions centered on the peak, and arc I does not exist.

Figure 4 shows a close up view of the same left thigh pitch trace during the sit-to-stand transition from Figures 3A,B, along with the peak attributes and time points used to derive the features for these movements. A complete description of the arcs is provided in the Supplementary Materials. Arcs A-H correspond to the sit-to-stand transition, while arcs J-R correspond to the same attributes during the stand-to-sit transition (there is no arc I). Arcs E and N (not shown) correspond to a 1-s time period centered around the maximum (i.e., peak of the arc) of the SI-ST transition (arc E) and ST-SI transition (arc N). The peak (shown here as a black circle) is bracketed by the step end (to the right, black square) and the start of the rise (to the left, dark blue triangle). To avoid eccentricities arising from false starts and additional partial movements, the start of calculations is sometimes represented by the 20% rise point (cyan diamond, left), and the 80% return point (cyan circle, right).

### Features of SI-ST and ST-SI transitions: repeatability

Before determining which features were most likely to be affected in our cohort by MS, we sought to determine which of the features were clearly repeatable. Because each of the participants performed the TUG task twice, we compared the value of each feature during the first attempt and the second attempt. We analyzed the correlation using the Intraclass Correlation Coefficient (ICC). The features we tested were based on the pitch angular velocity measurements from both thighs and the spine sensor, roll angular velocity measurements from the spine sensor, a range of smoothness metrics, and an omnibus measure of TUG duration based on the Anterior-Posterior accelerometer of the thigh. The calculations were the absolute value (magnitude) of the peak angular velocity, the many possible durations of the event (as determined by the arcs as explained in the methods and Figure 4), the magnitude of the mean angular velocities for those arcs, the area under the curve for those arcs, and the smoothness of each arc (see Methods). Each pitch feature was initially calculated for both left and right thighs (and also for the spine), and the final thigh features were the maximum of the two thigh values, the minimum of the two thigh values, the value associated with the thigh making the first step, and the value associated with the thigh making the second step. In broad terms, we started with 819 features (many of which were highly related), of which 152 had an ICC ≥ 0.60 [a good correlation according to (58)].

**Figure 5:**
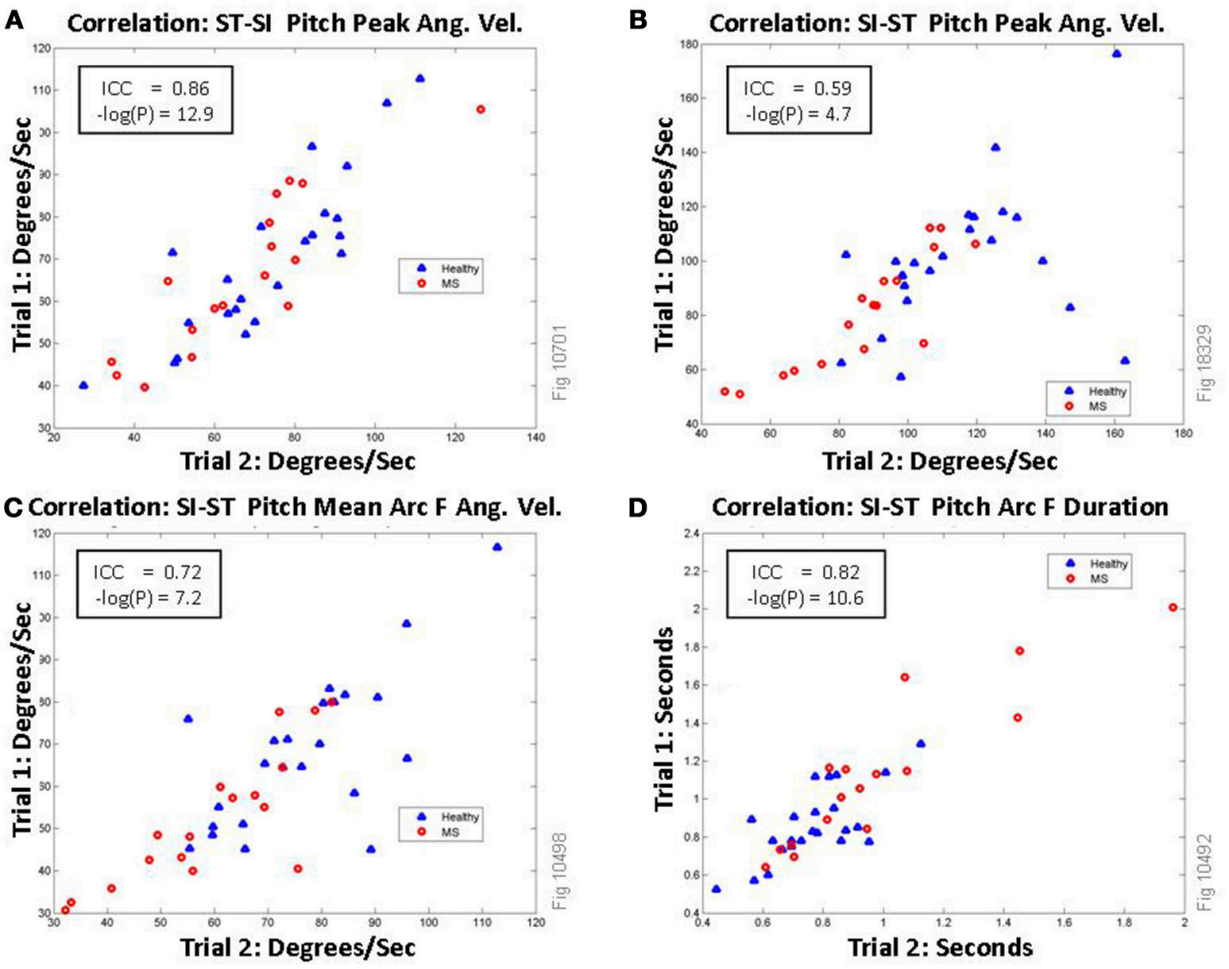
Correlations of selected features of thigh pitch signal (maximum of the left/right thigh) between trial 1 and trial 2 of TUG. Persons with MS are shown as red circles, healthy volunteers are shown as blue triangles. **(A)** shows excellent correlation between the two TUG trials each participant performed for the feature: the absolute value of the peak pitch angular velocity during the Stand-to-Sit (ST-SI) transition (magenta circle in Figure 3C). **(B)** This can be compared directly to the same measurement during the Sit-to-Stand (SI-ST) transition (magenta circle in Figure 3B), which shows only fair correlation. **(C)** shows the absolute value of the mean angular velocity of the signal (as shown as arc F in Figure 4) during the Sit-to-Stand transition. **(D)** shows the correlations for the duration of the sit-to-stand phase (arc F).

Representative plots showing selected correlations of four of the features are shown in Figure 5. The most correlated measurement arcs for the transitions are arc J, K, N, and M all of which encompass the entirety of the ST-SI peak (including the peak itself); the least correlated were arcs P and Q, both of which represent the first half of the ST-SI transition. The most consistent among the spine roll metrics are the SI-ST arcs that include the most possible time for unpredictable activity, including arcs B, F, E, and A, all of which had excellent correlations (ICC ≥ 0.75). The vast majority of smoothness metrics were poorly correlated, although a few were good (between 0.60 and 0.74). This may be expected, given that lack of smoothness would represent loss of control, which would per force be inconsistent.

### Features of SI-ST and ST-SI transitions: PwMS vs. healthy

**Figure 6:**
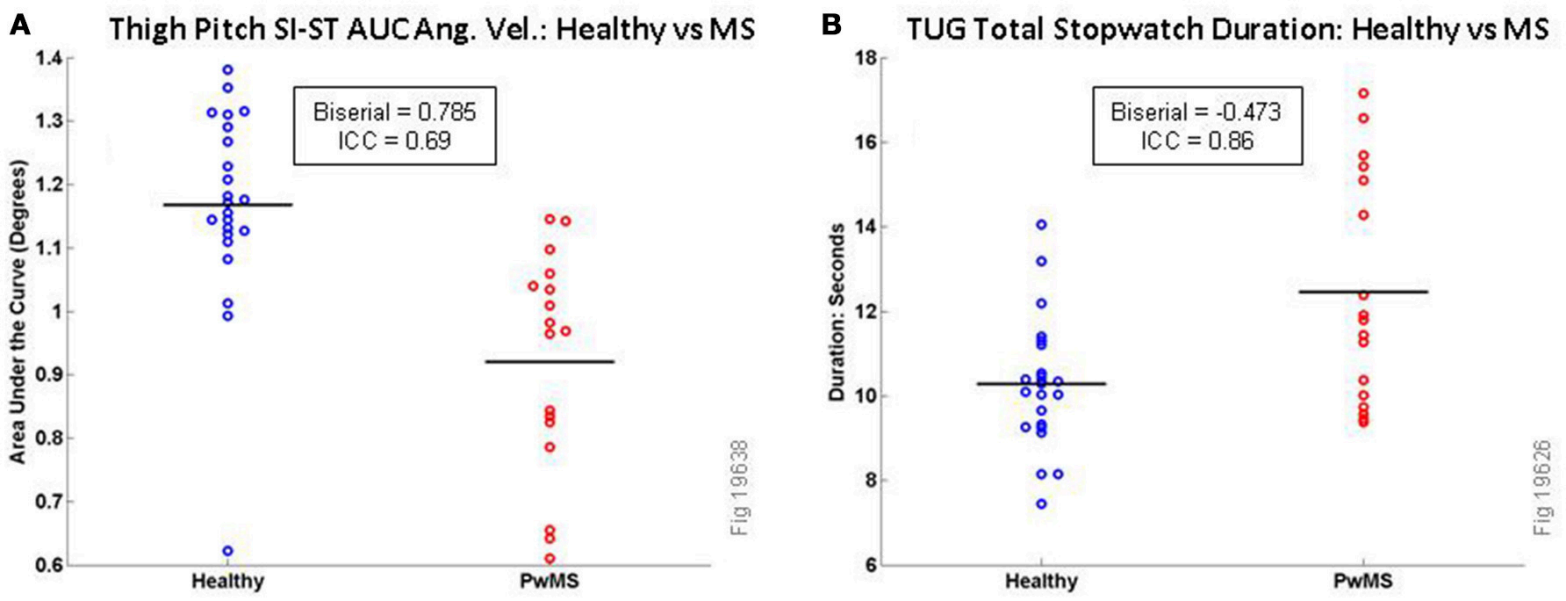
Comparison of TUG variables for Healthy vs. MS Participants for Area Under the Curve for SI-ST Thigh Pitch Angular Velocity (**A**, all values should be multiplied by 10^4^) and Total TUG Stopwatch Duration **(B)**. Black horizontal lines are mean values.

In total 819 correlated features were tested, and they were compared between the healthy volunteers and the PwMS. The raw *P*-values (Wilcoxon Rank Sum test) and the effect sizes (rank biserial) are shown in Table 2 for 27 of the most relevant TUG micro-features; a total of 134 features had raw *P* < 0.01. Those not included in the table were redundant or similar to other features already in the table [e.g., there were similar effect sizes for calculations based on the minimum (e.g., left or right thigh) and maximum]. To account for multiple comparisons, the Holm-Bonferroni method was used. Under this stringent method, only three features remained statistically significant, all of which related to SI-ST transition, based on the thigh pitch measurements of the area under the curve: feature 1 (Thigh Maximum Area Under the Curve for Arc B), feature 2 (Thigh Maximum Area Under the Curve for Arc D) and feature 3 (Thigh Maximum Area Under the Curve for Arc F). A comparison of feature 1 between Healthy and PwMS is shown in Figure 6A. The fastest 50% of healthy volunteers reach angular velocities that exceed all PwMS, while the slowest quartile of PwMS cannot reach angular velocities reached by all healthy volunteers (except for one healthy outlier, who was a tall (175 cm), middle-aged female who moved slowly and deliberately when getting in and out of the chair). To illustrate the scale of those differences, a comparison of the total TUG task durations (as measured by stopwatch) are shown next to this plot (see Figure 6B).

**Table 2:** List of selected features comparing healthy to PwMS.

**No**.	**Feature**	**Raw P rank sum**	**Rank biserial**	**Healthy median ± MAD**	**PwMS median ± MAD**	**ICC**
A	Total TUG duration thigh accel ant-post	0.01606	−0.453	9.14 ± 0.68	11.11 ± 2.27	0.86
B	Total TUG duration stopwatch	0.01183	−0.473	10.27 ± 1.53	12.44 ± 2.70	0.86
**THIGH PITCH SIT-TO-STAND (ANGULAR VEL)**						
1	Thigh maximum arc B area under curve (abs)	0.00003	0.785	11,720 ± 620	9,685 ± 1,292	0.69
2	Thigh maximum arc D area under curve (abs)	0.00004	0.775	11,634 ± 859	9,552 ± 1,309	0.62
3	Thigh maximum arc F area under curve (abs)	0.00005	0.760	11,448 ± 803	9,519 ± 1,244	0.61
4	Thigh maximum sit-to-stand peak (abs)	0.00030	0.678	117.91 ± 15.62	90.06 ± 15.07	0.74
5	Thigh second step arc F mean (abs)	0.00069	0.637	73.75 ± 9.22	55.12 ± 8.84	0.78
6	Thigh maximum arc F mean (abs)	0.00084	0.627	79.66 ± 9.59	63.44 ± 10.36	0.79
7	Thigh minimum arc F mean (abs)	0.00103	0.616	71.10 ± 11.27	55.12 ± 8.24	0.74
8	Thigh first step sit-to-stand peak (abs)	0.00103	0.616	110.09 ± 13.57	90.06 ± 15.07	0.59
9	Thigh minimum arc D mean (abs)	0.00165	0.591	69.85 ± 9.50	53.38 ± 7.57	0.71
10	Thigh second step sit-to-stand peak (abs)	0.00444	0.535	100.34 ± 14.66	86.35 ± 12.73	0.77
**THIGH PITCH STAND-TO-SIT (ANGULAR VEL)**						
11	Thigh maximum arc K area under curve (abs)	0.00239	0.570	8,958 ± 1,026	7,137 ± 338	0.50
12	Thigh maximum arc M area under curve (abs)	0.00373	0.545	8,616 ± 686	6,931 ± 430	0.51
13	Thigh minimum stand-to-sit peak (abs)	0.09513	0.315	68.73 ± 7.40	60.01 ± 12.53	0.85
**SPINE PITCH (ANGULAR VEL)**						
14	Spine pitch sit-to-stand peak 2 (abs)	0.00676	0.509	79.02 ± 11.37	60.92 ± 11.36	0.79
15	Spine pitch sit-to-stand peak 1 (abs)	0.00796	0.499	110.48 ± 14.30	83.21 ± 10.40	0.70
16	Spine pitch stand-to-sit peak 1 (abs)	0.07994	−0.330	70.84 ± 8.66	62.65 ± 12.46	0.56
17	Spine pitch arc Q AUC	0.44364	−0.146	-3,355 ± 825	−2,906 ± 914	0.66
**DURATIONS ALL**						
18	Thigh maximum arc F duration	0.01858	−0.442	1.19 ± 0.09	1.42 ± 0.15	0.62
19	Thigh minimum arc D duration	0.03871	−0.389	0.81 ± 0.13	0.95 ± 0.16	0.78
20	Spine sit-to-stand Weiss duration	0.13923	−0.279	0.70 ± 0.12	0.77 ± 0.19	0.67
21	Spine stand-to-sit Weiss duration	0.15867	−0.266	1.01 ± 0.10	1.22 ± 0.32	0.54
**SPINE ROLL (ANGULAR VEL)**						
22	Spine roll arc D smoothness 2	0.00675	0.509	−1.243 ± 0.100	−1.413 ± 0.141	0.53
23	Spine roll arc J mean (abs)	0.06679	−0.345	5.80 ± 1.84	8.69 ± 2.92	0.49
24	Spine roll arc B mean (abs)	0.07534	−0.335	15.29 ± 3.69	21.34 ± 8.76	0.80
25	Spine roll stand-to-sit peak 1 (abs)	0.07534	−0.335	30.75 ± 10.08	43.02 ± 15.07	0.75
26	Spine roll stand-to-sit peak 2 (abs)	0.11888	−0.294	15.77 ± 6.53	21.09 ± 5.16	0.40
27	Spine roll arc N smoothness 1	0.28596	0.202	−0.0372 ± 0.0093	−0.0423 ± 0.0172	0.09

*All angular velocities refer to pitch unless stated as roll. The features in each category are listed in order of the effect size (rank biserial); note that some features (13, 16, 17, 22, 23–27) are included as illustrative rather than as discriminatory features. 819 features were tested, so that with a Holm-Bonferroni method for multiple comparisons, only features 1, 2, and 3 (Area Under the Curve for arcs B, D and F) are significant. Arcs are as listed in Figure 4*.

When comparing the effect sizes (Rank Biserial in Table 2) of MS in our cohort of the features, several observations arise. The features relating to the sit-to-stand transition have a larger effect size (and are more consistently relevant when discriminating PwMS from healthy volunteers) than the stand-to-sit transition. The angular velocity features (Area under the Curve, absolute peak and absolute mean) have larger effect sizes (and are more consistently relevant when observing PwMS) than the durations. In our hands, the effect sizes of the durations arising from the spine sensor [features 21 and 22 in this study, originally from Weiss et al. (52)] have a smaller effect size than the homologous features measured with thigh sensors; furthermore, spine pitch peak angular velocity features (features 14–16) have larger effect sizes than spine duration features (features 20 and 21).

In our hands, in a univariate analysis roll of the spine sensor features had low rank biserials compared to the other tested features; the exception was for smoothness features, four of which had *P* < 0.05, including feature 22 (Spine Roll Arc D smoothness 2). As stated above, smoothness features were less consistent than other features. Among non-smoothness features derived from the roll of the spine sensor, the largest effect size of MS was on the mean of the angular velocity during ST-SI (arc J), which was associated with a raw *P* = 0.067 (rank biserial = −0.345).

### Multivariate analysis with logistic regression

As an unplanned analysis, we sought to understand how these variables might work together, given that many of the features were based on similar or related measurements. Using a stepwise procedure (Matlab), we removed variables that were weak contributors (low absolute *t*-values) or were not robust when subsets of volunteers were selected for the model. A set of seven features were found and described in a logistic regression (see Table 3). The regression had an *R*^2^ [coefficient of discrimination (59)] of 0.4708 based on 73° of freedom for error. None of the pairs of variables had a coefficient of correlation above 0.69 (Table 4). To check for overfitting, combined data for healthy and PwMS volunteers were randomly split in half (training set), betas were re-derived for the seven robust features, and the remaining volunteers (test set) were compared to predicted values based on the new betas; in 100 attempts, the average correct prediction rate was 0.7982. This implies that these features may be consistent enough to be useful in assessing degrees of mobility/disability among MS patients.

**Table 3:** Logistic regression to discriminate healthy from MS.

**Feat. No**.	**Sensor**	**Direction**	**Time**	**Arc**	**Calculation**	**Beta**	**S.E**.	***t***	***p***
3	Thigh	Pitch	SI-ST	Arc F	AUC	1.0666e-03	3.34579e-04	3.188	0.0026
14	Spine	Pitch	SI-ST	Peak 2	Abs	0.17734	0.05628	3.151	0.0020
B	Stopwatch		Complete	TUG	Duration	−1.2315	0.3996	−3.082	0.0090
22	Spine	Roll	SI-ST	Arc D	Smoothness 2	−92.326	33.041	−2.794	0.0093
26	Spine	Roll	ST-SI	Peak 2	Abs	−0.10683	0.03984	−2.681	0.0103
17	Spine	Pitch	Turn 2	Arc Q	AUC	−9.3272e-04	4.4917e-04	−2.077	0.0086
27	Spine	Roll	ST-SI	Arc N	Smoothness 1	46.015	22.486	2.046	0.0209
	Constant					−10.149	5.322	−1.907	0.0099

*For betas and t values that are positive, an increase in the feature's value implies the volunteer is healthy, while for negative values t and beta, higher values of the feature suggest that the volunteer is a person with MS. Rows are ordered by absolute value of t statistic, with the most contributory (and consistently discriminatory) features at the top. SI-ST, sit-to-stand transition; ST-SI, stand-to-sit transition; AUC, area under the curve; Abs, absolute value*.

**Table 4:** Correlation coefficients for logistic regression variables.

**Feat. No**.	**Feature name**	**Feat. 3**	**Feat. 14**	**Feat. B**	**Feat. 22**	**Feat. 26**	**Feat. 17**	**Feat. 22**	**Constant**
3	Pitch thigh AUC arc F	1							
14	Spine SI-ST P peak 2	0.6497	1						
B	TUG stopwatch	−0.5944	−0.6788	1					
22	Spine roll arc D smoothness 2	−0.4708	−0.6108	0.6115	1				
26	Spine roll ST-SI peak 2	−0.3076	−0.4255	0.6184	0.6541	1			
17	Spine pitch arc Q AUC	−0.4127	−0.3760	0.4943	0.5171	0.5824	1		
27	Spine roll arc N smoothness 1	0.3912	0.5824	−0.4617	−0.6010	−0.3227	−0.1667	1	
	Constant	−0.6858	−0.6106	0.0647	0.3809	−0.0138	0.3331	−0.2289	1

*SI-ST, sit-to-stand transition; ST-SI, stand-to-sit transition; AUC, area under the curve; Abs, absolute value*.

## Discussion

Inertial sensor metrics of gait and mobility variables, and their responsiveness to clinical conditions, are being explored for the differences elicited by sensor placement on different parts of the body (60). In this study of MS, we considered myriad TUG features (derived from previous studies of ambulatory disabilities of all kinds), and found informative metrics derived from thigh-positioned wearable inertial sensors that would be useful for estimating disability in PwMS, particularly with regard to strength and effort. We also compared a range of the best of the thigh-based metrics to spine-based metrics (which represent both strength and control), and ran a logistic regression on the results. We list seven non-overlapping features that may be useful together as complementary metrics in assessments of disability progression in MS, and also as metrics for clinical efficacy for interventions proposed to improve or limit disability in MS. In the present study, the test for whether these features may be useful for estimating disease progression was a comparison of a small community sample of PwMS with Hauser Ambulation Index scores ≤ 2 against a sample of middle-aged, healthy volunteers. Our novel contribution is to consider the combination of thigh and spine metrics in MS–as did Motta et al. (17) during a 1-min walking task. Our data specifically considers the case of TUG, which includes the SI-ST and ST-SI transitions; these transitions are particularly challenging activities in everyday life, and are especially revealing of the movement of the thigh segment.

As expected, we found that the total time duration of the TUG task as measured by stopwatch was a consistent and discriminatory feature (rank biserial = −0.473, *P* < 0.05) for these two cohorts; this is similar to a study of TUG in the elderly [Instrumental Activities of Daily Living (IADL) vs. no IADL] in which TUG duration was the most discriminatory feature (52), and to an MS vs. healthy comparison of the Timed 25 Foot Walk where overall velocity (which is usually measured as a stopwatch duration) was the most discriminatory mobility feature (53). In our cohorts we compared a wide variety of sensor-based micro-features of TUG to two timing features of TUG as a whole; we found that many of the thigh-derived sensor micro-features are reproducible and have high reliability, and that a collection of thigh pitch angular velocity features (including absolute values of the area under the curve, the peak and the mean) based on the sit-to-stand transition differed between MS and healthy with higher effect sizes (rank biserial) than total time duration of TUG; three of these features were statistically significantly different (between healthy and PwMS) by the stringent Holm-Bonferroni method of multiple comparisons. These features were all similar measurements of the area under the curve for pitch angular velocity for the SI-ST transition. Because the SI-ST transition is a demanding task for the musculature, and higher values for pitch angular velocity would be particularly demanding, we associate these variables with strength (28). This fits with previous research on patients with total knee arthroplasty that concluded that quadriceps weakness has a substantial impact on performance of the sit-to-stand task (20, 61).

We also tested temporal duration features based on the thigh SI-ST transition and previously published features based on the spine-derived SI-ST transition (52), and we found the set of such spine-derived features that were potentially useful, but those features resulted in lower effect sizes than the traditional stopwatch duration of TUG for our cohorts (and thus had lower effect sizes than the best angular velocity features). For both sit-to-stand and stand-to-sit transitions, spine data is discriminatory, but thigh data is more discriminatory for MS disability. We also measured many features suggesting that thigh pitch (or spine pitch) is much more discriminatory than spine roll.

Some previous studies have found discriminatory features within the roll of the spine (37), within the stand-to-sit transition (26, 33, 62), and from jerk-related smoothness of angular velocity signals (21), all of which would reflect diminished balance and control rather than strength/weakness. In our cohorts these types of features produced smaller univariate effect sizes, and those roll features that were reliable (ICC) did not reach raw *P*-values under *P* < 0.05 (except for feature 22).

In a logistic regression we found that our initial hypothesis was supported: the movement of the thigh during the SI-ST transition was the most informative of all the TUG measures tested, and that adding a thigh feature (feature 3) robustly improved a logistic regression compared to using only spine features with the total TUG duration. However, we were surprised to find that five of the seven robust features were from the spine sensor, three were related to roll, and two were related to smoothness; none of the other thigh features were independent or robust enough to stay in the analysis after the first one was included. Of the spine features, it is intuitive that healthy volunteers have a large pitch SI-ST peak (feature 14, implying torso strength and effort), and that PwMS have a larger roll peak during ST-SI (feature 26, implying loss of torso control). It also makes some sense that healthy volunteers would have a smoother roll in angular momentum in the 1 s surrounding the ST-SI peak (feature 27, arc N, Figure 4). It was interesting to find that the PwMS had a larger AUC of spine pitch in arc Q (feature 17); arc Q is the first half of the ST-SI transition, and when picked by our algorithm is made up primarily of Turn 2 of the TUG. It is less intuitive that the spine roll signal during most of the SI-ST transition (feature 22, arc D) would be smoother for MS patients than for healthy volunteers; presumably this relates to MS patients being slower and more cautious when rising (using the chair's arms), but none of the other calculations (peak, mean or duration) is discriminatory in this way.

This hierarchy of discriminatory power (strength > control) seems to be supported by some other studies working on other ambulatory disorders. A previous study examining the shank-mounted sensor metrics of TUG (as an entire task) in PwMS (16) found that their regression models for clinical disability metrics [EDSS and Multiple Sclerosis Impact Scale (MSIS-20)] incorporated many sensor metrics of angular velocity including mean angular velocities, maximum angular velocities, and minimum (i.e., trough negative) angular velocities (all multiplied by patient height), while it rejected coefficients of variation, and many gait duration features (e.g., mean stride time, mean swing time, mean double support %, turning time). In a study of the elderly (33, 52), the range of the vertical accelerometry signal (located at the lumbar spine) was a discriminatory feature for identifying idiopathic fallers among the elderly, while SI-ST duration and ST-SI duration were not discriminatory.

### Relevance of sensor assessment of mobility in the clinic

The use of inertial sensor technology in clinical assessment of disability is moving ahead rapidly in both MS and in disorders of mobility more generally. The goal of such systems is to increase the resolution and consistency of measurements of ambulatory disability (e.g., might it be possible to consistently recognize a difference between an ambulatory equivalent of EDSS 4.2 vs. EDSS 4.3). Only further sensor research on clinical populations will clarify whether this goal is even possible. Currently a commercial system for measuring mobility during TUG that is operated by clinicians (i.e., not researchers or engineers) has been released and assessed by the UK's National Institute for Health and Care Excellence (63). Extensive research into this particular inertial sensor methodology has been driven by the manufacturer of this system, which places sensors near the ankles. In a cross-sectional study of early stage relapsing remitting MS, the ankle-based sensor system used a proprietary algorithm to produce an EDSS estimate that was shown to correlate moderately well (*R*^2^ = 0.5) with clinician assessed EDSS (16). More recently the same system was able to predict the 90-day risk of falls of Parkinson's patients with a 73% accuracy during a 6 month longitudinal trial (64).

### Analysis details

The most clear result here is that for univariate associations, the hierarchy of discrimination is broadly: area under the curve > mean/peak angular velocity > duration. This dominance by AUC was slightly unexpected, as mean/peak velocity features might be expected to vary inversely with duration measures; however, when thinking about the entire movement, duration multiplied by movement is a more comprehensive measure of the total effort and strength than the peak (or the mean) is. It is worth noting that the ICC for AUC features were generally not as high as for peak or mean features. Duration features were quite variable.

The rationale for positioning wearable inertial sensors on the thighs for characterizing the sit-to-stand and stand-to-sit transitions is that the activity of the thighs during these transitions is invariably both necessary and sufficient to achieve these actions, while the activity of the spine and torso are usually necessary but are definitely not sufficient. For example, additional torso activity may occur during bodily adjustments or false starts, and torso activity can be suppressed while rising up or sitting down with the use of the chair's arms. Nevertheless, our regression favored spine metrics.

Regarding false starts and bodily adjustments, it is slightly easier to detect the difference between healthy and PwMS from overall absolute peak angular velocity values or from means derived from time segments that do not include the bottom 20% of activity (i.e., arc F on Figure 3B has a higher effect size than arc B). The values for pitch angular velocity are higher for healthy than for MS; the regions of the bottom 20% of activity may be associated with brief, abortive initiations of standing, which are inconsistent but common to both healthy and mild MS, thus masking the appropriate durations or mean values of the transitions. Note also that the calculations of durations are made less valid (lower absolute effect size) by including the bottom 20% of activity; the rank biserial for SI-ST duration (maximum from either thigh) when based on Arc F (which does not include the lower 20%, see Figure 4) is −0.442, compared to the rank biserial for the same value based on Arc B is −0.358.

By contrast, for area under the curve measures, where increased duration adds to the appearance of strength in the healthy participants, the bottom 20% of the curve adds slightly to the discrimination between MS and healthy (i.e., arc B has a greater absolute effect size compared to arc F). In general strength measurements based on angular velocity had higher discriminatory power if the maximum of the two thighs was used (compared to the lesser value from the two thighs). Also, for spine roll features, where MS is associated with higher values of roll angular velocity than seen in healthy volunteers, this increased roll is easier to detect in longer segments that include the bottom 20% of the entire peak region.

### Limitations

One limitation of the current study is that we did not make concurrent measurements of strength (e.g., the Oxford Scale for Muscle Strength Grading), nor did we estimate spasticity (e.g., Modified Ashworth Scale); plainly there are differences in the types of MS mobility impairment (65), and there would be a difference in the test results between a PwMS with flaccid paralysis vs. a PwMS with normal strength and a high level of spasticity. In future measurements of the SI-ST transition, measurements of strength and spasticity should accompany sensor measurements, as this is often not done (16, 66).

Another limitation is that for inertial sensor metrics to be justified for use in the clinic to assess disability or mobility impairment, a longitudinal study needs to be performed. Such a longitudinal study would ideally show that clinically relevant disability progression (or amelioration due to therapeutic intervention) could be detected with more sensitivity and consistency by the sensor metrics than by the EDSS (or possibly by the MSFC). Recognising fine-grained differences against a “gold standard” measurement such as the EDSS will require an agreement or recognition as to how to recognize (or cause) small changes in disability independently of the EDSS.

Inconsistency between equally disabled patients (or between measurements from the same patient on different days) may affect many individual metrics because patients may compensate for their disability with additional motivation; it would be expected that when this compensation occurs, there would be a deterioration of performance control (e.g., spine roll during TUG) because of the speed-accuracy trade-off (67, 68). When considering speed and limb movements during walking tasks (e.g., T25FW), motivation (or lack thereof) can affect walking speed; however, lack of motivation alone will be less likely to affect peak angular velocity during the SI-ST transition, because standing up slowly requires more prolonged effort than standing up quickly, due to the disadvantageous torque moments that have to be resisted during slow standing (69).

The sensors used during this study were recorded independently and were later synchronized using an automated synchronization protocol. While this produces accurate data synchronization, it prevents real-time analysis, which would be essential for clinical use. Since the gathering of this data, the manufacturer of the sensors (x-io) has introduced a new generation of IMU sensors (NGIMU), which include WiFi communication and the use of one sensor as a master sensor to calibrate all others on the network (70). In the future, these self-synchronizing sensors should be used for gathering data.

In our regression, we found a few features with smaller effect sizes (many of which are more related to accuracy/control rather than speed/strength) that may be relevant for estimating disability in PwMS, particularly when assessing PwMS who have mild or almost no ambulatory dysfunction. Likewise, the many uncorrelated features rejected from the final list of features may include some usefully discriminating features that could be used as metrics of balance and control during movement.

The generalizability of these results for PwMS may be limited due to the precise nature of the TUG task format, as well as due to the idiosyncrasies of PwMS. For example, Boonstra et al. (20) used a special sit-to-stand assay that differed from the TUG in several important aspects; their chair did not have arms, their arthroplasty patients had to position their hands on their hips so that they could not use their arms to aid in standing, and the task did not continue directly into a walking task. Another feature of their protocol that differed from the current study is that their chair had an adjustable chair height so the participants' knees always started at 90°. The precise position of the knees at the beginning of rising will affect measurements of activity, especially angular velocity. In the TUG protocol the participant is allowed to start with their legs in self-selected positions, which would mean that the first movement during TUG would include repositioning of the lower limb into an optimum position for the sit-to-stand transition.

## Conclusions

Our data suggest that positioning sensors on the thighs and measuring pitch angular velocities during the sit-to-stand transition can provide information relating to disability in multiple sclerosis that is more relevant (with larger effect sizes) than both (a) durations of sit-to-stand derived from a lumbar spine sensor, and (b) durations of the entire TUG task. Our data suggests that adding a thigh sensor-based metric can increase discriminatory power compared to using a spine sensor alone, and that for mild to modest disability (HAI ≤ 2), features that reflect weakness (or strength) are more discriminatory than features that reflect loss of control or imbalance. Finally, the area under the curve, the peak and mean angular velocities, the durations, and the roll measures may provide more universal and broadly-sensitive information if they are combined into a composite metric, although for any such metric to be adopted by the medical community, it would have to be transparent. Our regression data included the SI-ST transition, ST-SI transition, part of Turn 2, and overall gait performance (TUG stopwatch time), all of which were contributory to the model.

## Author contributions

HW: idea, obtain funding, study design, supervision (primary), equipment provision, analysis (primary), drafting manuscript (primary). CO: data gathering (primary), analysis. RN: data gathering, analysis. AH: data gathering, analysis, editing. CW: analysis. JG: analysis, data gathering. JaB: data gathering. JeB: supervision, equipment provision. CH: equipment provision. DR equipment provision, supervision. BE: idea, obtain funding, study design, supervision. WR: idea, obtain funding, study design, equipment provision. NC: equipment provision, supervision. JK: idea, obtain funding (primary).

### Conflict of interest statement

JeB and CH were employees of ASTRUM IT GmbH at the time of this study. ASTRUM IT manufactures a gait system, but not the system used in this study. The remaining authors declare that the research was conducted in the absence of any commercial or financial relationships that could be construed as a potential conflict of interest.

## Abbreviations

Ang. Vel.: angular velocity
AUC: area under the curve
BDI: beck depression scale
csv: comma separated variables format of data file
EDSS: expanded disability and status scale
EDSS-S: self-assessed version of the EDSS
FSS
fatigue severity scale
HAI: Hauser ambulatory index
ICF: International Classification of Functioning
IMU: inertial motion unit
IPAQ: International Physical Activity Questionnaire
L3: Lumbar vertebra 3
MEMS: micro-electromechanical sensor
MFIS: modified fatigue impact scale
MS: multiple sclerosis
MSFC: multiple sclerosis functional composite
MSWS-12, multiple sclerosis walking scale: 12 item version
PwMS: persons with multiple sclerosis
RRMS: relapsing remitting multiple sclerosis
SI-ST: sit-to-stand transition during TUG
ST-SI: stand-to-sit transition during TUG
T25FW: timed-25-foot walk
TUG: timed up and go test.
