# Supplementary material for: Thigh-Derived Inertial Sensor Metrics to Assess the Sit-to-Stand and Stand-to-Sit Transitions in the Timed Up and Go (TUG) Task for Quantifying Mobility Impairment in Multiple Sclerosis
Source: Front Neurol. 2018 Sep 14;9:684. doi: 10.3389/fneur.2018.00684 (PMC6149240; doi:10.3389/fneur.2018.00684)
Supplement: Supplementary file 1 [file Data_Sheet_1.doc]

## Supplementary Methods

### Appendix 1: Explanation of the Arcs Analysed as Shown in Figure 04


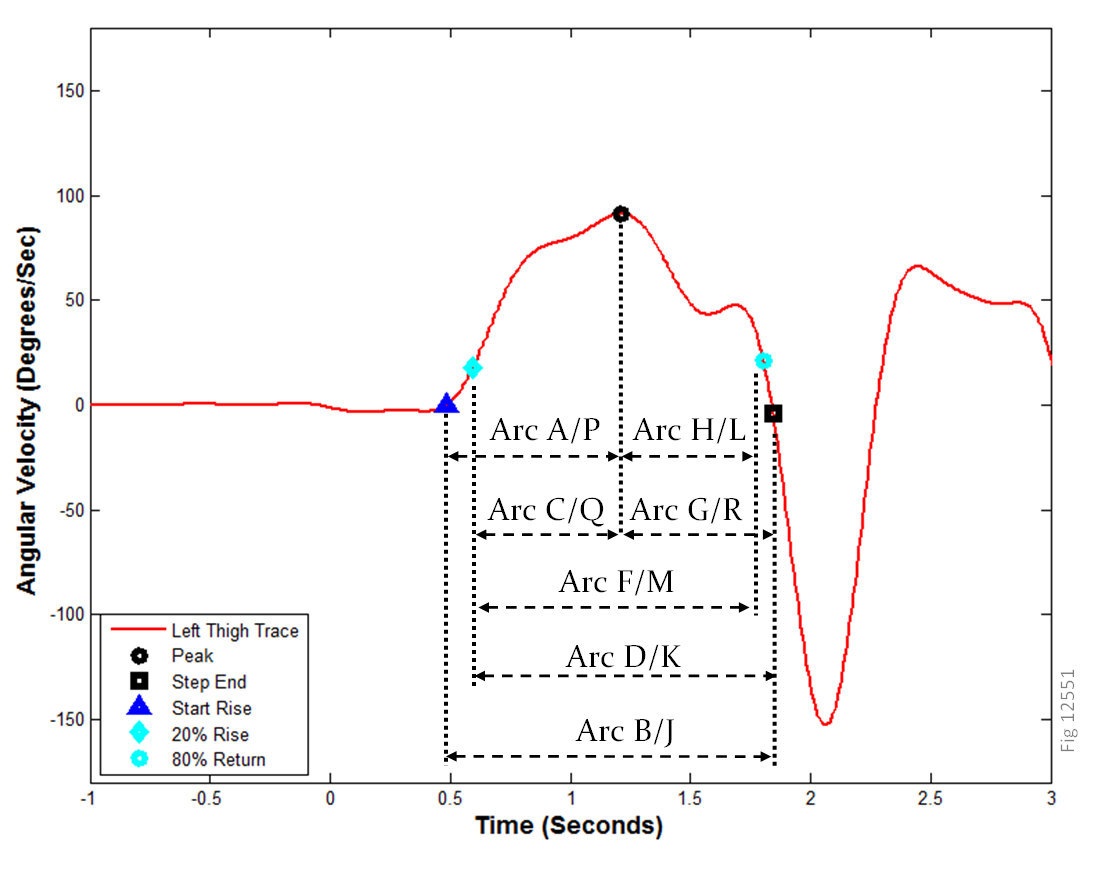


Arcs A-H correspond to the SI-ST transition, while arcs J-R represent the ST-SI transition. The peak (shown here as a black circle) is bracketed by the step end (to the right, black square) and the start of the rise (to the left, blue triangle). To avoid eccentricities arising from false starts and additional partial movements, the start is sometimes represented by the 20% rise point (cyan diamond, left), and the 80% return point (cyan circle, right). The most consistent and distinguishing calculations relate to the sit-to-stand elements arc C (from 20% rise point to the peak/trough), arc F (20% rise point to 80% return point), and arc D (20% rise point to step end). How these points were computationally derived is described in the methods; note that arcs E and N (not shown) are 1 second regions centred on the peak, and arcs I and J were not calculated.

| ArcTiming | Explanation |
| --- | --- |
| Arc A  SI-ST | Early thigh SI-ST activity. Starts with the initial angular acceleration of the thigh and ends when the thigh's angular acceleration is at the peak. The end can be consistently detected while the beginning may be contaminated by activity preceding the actual act of standing. The potential advantage of measuring early SI-ST activity over complete SI-ST activity (see arc B) is that the disadvantageous leverage during early SI-ST strongly discourages slowness or pausing, while the better leverage during the latter half of SI-ST allows for poor motivation to affect performance. |
| Arc B  SI-ST | Complete thigh SI-ST activity to first step. Starts with the initial angular acceleration of the thigh and ends when the thigh's angular acceleration is at the zero crossing point. The end can be usually detected while the beginning may be contaminated by activity preceding the actual act of standing. |
| Arc C  SI-ST | Clean early thigh SI-ST activity. Starts with the initial angular acceleration of the thigh (delayed to 20% rise point) and ends when the thigh's angular acceleration is at the peak. Both the end and the start can be consistently detected. This was expected to be a more reliable period for automated analysis than A. The advantage of starting at the 20% launch point is that the initiation of activity (blue triangle) often relates to movements that precede standing up (such as adduction of the thighs); these preparatory movements can represent false starts and may not relate to the SI-ST activity. By starting at the 20% rise point (cyan diamond), it guarantees that the measurements are during the true SI-ST, when the buttocks have left the seat of the chair. |
| Arc D  SI-ST | Cleaned complete thigh SI-ST activity to first step. Starts with the initial angular acceleration of the thigh and ends when the thigh's angular acceleration is at the zero crossing point. The beginning can be reliably detected while the end can be usually detected. |
| Arc E  SI-ST | 1-Second Activity Surrounding Peak (not shown). This arc reflects the peak and the broadness of the peak. The peak is the maximum angular acceleration that occurs during the SI-ST action. The peak is consistently identified, and the 0.5 seconds on either side of the peak always can be found. |
| Arc F  SI-ST | Cleaned complete thigh SI-ST activity to first step. Starts with the initial angular acceleration of the thigh (delayed to 20% rise point) and ends when the thigh's angular acceleration is at the zero crossing point (advanced to 80% return point). Both the end and the beginning should be able to be detected without contamination. The advantage of using the 80% return point is that sometimes the second half of the curve does not go directly down to the zero crossing point but has a "wiggle" (you can see a small wiggle on this curve). The 80% return point sometimes prevents wiggles from extending the curve unnecessarily. After analysis of our data, it appears that this distinction (i.e. between arc D and arc F) is not very important. |
| Arc G  SI-ST | Late thigh SI-ST activity to first step. Starts with the peak angular acceleration of the thigh and ends when the thigh's angular acceleration is at the zero crossing point. Both the beginning and the end can be reliably detected. |
| Arc H  SI-ST | Cleaned late thigh SI-ST activity to first step. Starts with the peak angular acceleration of the thigh and ends when the thigh's angular acceleration is at the zero crossing point. Both the beginning and the end can be reliably detected. |
| Arc I | Not measured. |
| Arc J  ST-SI | Complete thigh ST-SI activity from the final step to being seated. In the TUG the ST-SI transition detected by the thigh includes three actions: the final step, the 180 degree rotation, and then the ST-SI transition proper. Starts with the initial angular acceleration of the thigh and ends when the thigh's angular acceleration is at the zero crossing point. The end can be usually detected (although it sometimes has transient reversals that can interfere) while the beginning is often contaminated by inconsistency of the final step before the turn. |
| Arc K  ST-SI | Complete thigh ST-SI activity from just before the final step to being seated. Starts just after the initial angular acceleration of the thigh (20% rise point) and ends when the thigh's angular acceleration is at the zero crossing point. The end can be usually detected while the beginning is often contaminated by inconsistency of the final step before the turn. |
| Arc L  ST-SI | Late thigh ST-SI activity from the peak to just before being seated. The peak at the start of this arc is the peak of the proper ST-SI transition (i.e. after the 180 degree turn) and ends just before the thigh's angular acceleration is at the zero crossing point (80% return point). Both the start and the end are reliably detected. |
| Arc M  ST-SI | Cleaned complete thigh ST-SI activity from just before the final step to just before being seated. Starts just after the initial angular acceleration of the thigh (20% rise point) and ends just before the thigh's angular acceleration is at the zero crossing point (80% return point). The end can be usually detected while the beginning is sometimes contaminated by inconsistency of the final step before the turn. |
| Arc N  ST-SI | 1-Second Activity Surrounding Peak (not shown). This arc reflects the peak and the broadness of the peak. The peak is the maximum angular acceleration that occurs during the ST-SI action. The peak is consistently identified, and the 0.5 seconds on either side of the peak always can be found. |
| Arc P  ST-SI | Early thigh ST-SI activity. Starts with the initial angular acceleration of the thigh and ends when the thigh's angular acceleration is at the peak. The end can be consistently detected while the beginning is almost always contaminated by activity preceding the last step before the turn. |
| Arc Q  ST-SI | Cleaned early thigh ST-SI activity. Starts with the initial angular acceleration of the thigh and ends when the thigh's angular acceleration is at the peak. The end can be consistently detected while the beginning is usually contaminated by activity preceding the last step before the turn. |
| Arc R  ST-SI | Late thigh SI-ST activity to first step. Starts with the peak angular acceleration of the thigh and ends when the thigh's angular acceleration is at the zero crossing point. Both the beginning and the end can be reliably detected. |

### Appendix 2: List of Features

| ROTATION | BODY PART | SIDE | SEGMENT | CALCULATION |
| --- | --- | --- | --- | --- |
| none | thigh | right | accelY | duration |
| none | thigh | left | accelY | duration |
| none | stopwatch | TUG | complete | duration |
| pitch | thigh | firstStep | arcA | AreaUnderCurve |
| pitch | thigh | left | arcA | AreaUnderCurve |
| pitch | spine | lumbar | arcA | AreaUnderCurve |
| pitch | thigh | maxima | arcA | AreaUnderCurve |
| pitch | thigh | minima | arcA | AreaUnderCurve |
| pitch | thigh | right | arcA | AreaUnderCurve |
| pitch | thigh | secondStep | arcA | AreaUnderCurve |
| pitch | thigh | firstStep | arcA | AverageUnderCurve |
| pitch | thigh | left | arcA | AverageUnderCurve |
| pitch | spine | lumbar | arcA | AverageUnderCurve |
| pitch | thigh | maxima | arcA | AverageUnderCurve |
| pitch | thigh | minima | arcA | AverageUnderCurve |
| pitch | thigh | right | arcA | AverageUnderCurve |
| pitch | thigh | secondStep | arcA | AverageUnderCurve |
| pitch | thigh | firstStep | arcA | duration |
| pitch | thigh | left | arcA | duration |
| pitch | spine | lumbar | arcA | duration |
| pitch | thigh | maxima | arcA | duration |
| pitch | thigh | minima | arcA | duration |
| pitch | thigh | right | arcA | duration |
| pitch | thigh | secondStep | arcA | duration |
| pitch | thigh | firstStep | arcA | mean |
| pitch | thigh | left | arcA | mean |
| pitch | spine | lumbar | arcA | mean |
| pitch | thigh | maxima | arcA | mean |
| pitch | thigh | minima | arcA | mean |
| pitch | thigh | right | arcA | mean |
| pitch | thigh | secondStep | arcA | mean |
| pitch | thigh | firstStep | arcA | smoothness |
| pitch | thigh | left | arcA | smoothness |
| pitch | spine | lumbar | arcA | smoothness |
| pitch | thigh | maxima | arcA | smoothness |
| pitch | thigh | minima | arcA | smoothness |
| pitch | thigh | right | arcA | smoothness |
| pitch | thigh | secondStep | arcA | smoothness |
| pitch | thigh | firstStep | arcA | smoothness2 |
| pitch | thigh | left | arcA | smoothness2 |
| pitch | spine | lumbar | arcA | smoothness2 |
| pitch | thigh | maxima | arcA | smoothness2 |
| pitch | thigh | minima | arcA | smoothness2 |
| pitch | thigh | right | arcA | smoothness2 |
| pitch | thigh | secondStep | arcA | smoothness2 |
| pitch | thigh | firstStep | arcB | AreaUnderCurve |
| pitch | thigh | left | arcB | AreaUnderCurve |
| pitch | spine | lumbar | arcB | AreaUnderCurve |
| pitch | thigh | maxima | arcB | AreaUnderCurve |
| pitch | thigh | minima | arcB | AreaUnderCurve |
| pitch | thigh | right | arcB | AreaUnderCurve |
| pitch | thigh | secondStep | arcB | AreaUnderCurve |
| pitch | thigh | firstStep | arcB | AverageUnderCurve |
| pitch | thigh | left | arcB | AverageUnderCurve |
| pitch | spine | lumbar | arcB | AverageUnderCurve |
| pitch | thigh | maxima | arcB | AverageUnderCurve |
| pitch | thigh | minima | arcB | AverageUnderCurve |
| pitch | thigh | right | arcB | AverageUnderCurve |
| pitch | thigh | secondStep | arcB | AverageUnderCurve |
| pitch | thigh | firstStep | arcB | duration |
| pitch | thigh | left | arcB | duration |
| pitch | spine | lumbar | arcB | duration |
| pitch | thigh | maxima | arcB | duration |
| pitch | thigh | minima | arcB | duration |
| pitch | thigh | right | arcB | duration |
| pitch | thigh | secondStep | arcB | duration |
| pitch | thigh | firstStep | arcB | mean |
| pitch | thigh | left | arcB | mean |
| pitch | spine | lumbar | arcB | mean |
| pitch | thigh | maxima | arcB | mean |
| pitch | thigh | minima | arcB | mean |
| pitch | thigh | right | arcB | mean |
| pitch | thigh | secondStep | arcB | mean |
| pitch | thigh | firstStep | arcB | smoothness |
| pitch | thigh | left | arcB | smoothness |
| pitch | spine | lumbar | arcB | smoothness |
| pitch | thigh | maxima | arcB | smoothness |
| pitch | thigh | minima | arcB | smoothness |
| pitch | thigh | right | arcB | smoothness |
| pitch | thigh | secondStep | arcB | smoothness |
| pitch | thigh | firstStep | arcB | smoothness2 |
| pitch | thigh | left | arcB | smoothness2 |
| pitch | spine | lumbar | arcB | smoothness2 |
| pitch | thigh | maxima | arcB | smoothness2 |
| pitch | thigh | minima | arcB | smoothness2 |
| pitch | thigh | right | arcB | smoothness2 |
| pitch | thigh | secondStep | arcB | smoothness2 |
| pitch | thigh | firstStep | arcC | AreaUnderCurve |
| pitch | thigh | left | arcC | AreaUnderCurve |
| pitch | spine | lumbar | arcC | AreaUnderCurve |
| pitch | thigh | maxima | arcC | AreaUnderCurve |
| pitch | thigh | minima | arcC | AreaUnderCurve |
| pitch | thigh | right | arcC | AreaUnderCurve |
| pitch | thigh | secondStep | arcC | AreaUnderCurve |
| pitch | thigh | firstStep | arcC | AverageUnderCurve |
| pitch | thigh | left | arcC | AverageUnderCurve |
| pitch | spine | lumbar | arcC | AverageUnderCurve |
| pitch | thigh | maxima | arcC | AverageUnderCurve |
| pitch | thigh | minima | arcC | AverageUnderCurve |
| pitch | thigh | right | arcC | AverageUnderCurve |
| pitch | thigh | secondStep | arcC | AverageUnderCurve |
| pitch | thigh | firstStep | arcC | duration |
| pitch | thigh | left | arcC | duration |
| pitch | spine | lumbar | arcC | duration |
| pitch | thigh | maxima | arcC | duration |
| pitch | thigh | minima | arcC | duration |
| pitch | thigh | right | arcC | duration |
| pitch | thigh | secondStep | arcC | duration |
| pitch | thigh | firstStep | arcC | mean |
| pitch | thigh | left | arcC | mean |
| pitch | spine | lumbar | arcC | mean |
| pitch | thigh | maxima | arcC | mean |
| pitch | thigh | minima | arcC | mean |
| pitch | thigh | right | arcC | mean |
| pitch | thigh | secondStep | arcC | mean |
| pitch | thigh | firstStep | arcC | smoothness |
| pitch | thigh | left | arcC | smoothness |
| pitch | spine | lumbar | arcC | smoothness |
| pitch | thigh | maxima | arcC | smoothness |
| pitch | thigh | minima | arcC | smoothness |
| pitch | thigh | right | arcC | smoothness |
| pitch | thigh | secondStep | arcC | smoothness |
| pitch | thigh | firstStep | arcC | smoothness2 |
| pitch | thigh | left | arcC | smoothness2 |
| pitch | spine | lumbar | arcC | smoothness2 |
| pitch | thigh | maxima | arcC | smoothness2 |
| pitch | thigh | minima | arcC | smoothness2 |
| pitch | thigh | right | arcC | smoothness2 |
| pitch | thigh | secondStep | arcC | smoothness2 |
| pitch | thigh | firstStep | arcD | AreaUnderCurve |
| pitch | thigh | left | arcD | AreaUnderCurve |
| pitch | spine | lumbar | arcD | AreaUnderCurve |
| pitch | thigh | maxima | arcD | AreaUnderCurve |
| pitch | thigh | minima | arcD | AreaUnderCurve |
| pitch | thigh | right | arcD | AreaUnderCurve |
| pitch | thigh | secondStep | arcD | AreaUnderCurve |
| pitch | thigh | firstStep | arcD | AverageUnderCurve |
| pitch | thigh | left | arcD | AverageUnderCurve |
| pitch | spine | lumbar | arcD | AverageUnderCurve |
| pitch | thigh | maxima | arcD | AverageUnderCurve |
| pitch | thigh | minima | arcD | AverageUnderCurve |
| pitch | thigh | right | arcD | AverageUnderCurve |
| pitch | thigh | secondStep | arcD | AverageUnderCurve |
| pitch | thigh | firstStep | arcD | duration |
| pitch | thigh | left | arcD | duration |
| pitch | spine | lumbar | arcD | duration |
| pitch | thigh | maxima | arcD | duration |
| pitch | thigh | minima | arcD | duration |
| pitch | thigh | right | arcD | duration |
| pitch | thigh | secondStep | arcD | duration |
| pitch | thigh | firstStep | arcD | mean |
| pitch | thigh | left | arcD | mean |
| pitch | spine | lumbar | arcD | mean |
| pitch | thigh | maxima | arcD | mean |
| pitch | thigh | minima | arcD | mean |
| pitch | thigh | right | arcD | mean |
| pitch | thigh | secondStep | arcD | mean |
| pitch | thigh | firstStep | arcD | smoothness |
| pitch | thigh | left | arcD | smoothness |
| pitch | spine | lumbar | arcD | smoothness |
| pitch | thigh | maxima | arcD | smoothness |
| pitch | thigh | minima | arcD | smoothness |
| pitch | thigh | right | arcD | smoothness |
| pitch | thigh | secondStep | arcD | smoothness |
| pitch | thigh | firstStep | arcD | smoothness2 |
| pitch | thigh | left | arcD | smoothness2 |
| pitch | spine | lumbar | arcD | smoothness2 |
| pitch | thigh | maxima | arcD | smoothness2 |
| pitch | thigh | minima | arcD | smoothness2 |
| pitch | thigh | right | arcD | smoothness2 |
| pitch | thigh | secondStep | arcD | smoothness2 |
| pitch | thigh | firstStep | arcE | AreaUnderCurve |
| pitch | thigh | left | arcE | AreaUnderCurve |
| pitch | spine | lumbar | arcE | AreaUnderCurve |
| pitch | thigh | maxima | arcE | AreaUnderCurve |
| pitch | thigh | minima | arcE | AreaUnderCurve |
| pitch | thigh | right | arcE | AreaUnderCurve |
| pitch | thigh | secondStep | arcE | AreaUnderCurve |
| pitch | thigh | firstStep | arcE | AverageUnderCurve |
| pitch | thigh | left | arcE | AverageUnderCurve |
| pitch | spine | lumbar | arcE | AverageUnderCurve |
| pitch | thigh | maxima | arcE | AverageUnderCurve |
| pitch | thigh | minima | arcE | AverageUnderCurve |
| pitch | thigh | right | arcE | AverageUnderCurve |
| pitch | thigh | secondStep | arcE | AverageUnderCurve |
| pitch | thigh | firstStep | arcE | duration |
| pitch | thigh | left | arcE | duration |
| pitch | spine | lumbar | arcE | duration |
| pitch | thigh | maxima | arcE | duration |
| pitch | thigh | minima | arcE | duration |
| pitch | thigh | right | arcE | duration |
| pitch | thigh | secondStep | arcE | duration |
| pitch | thigh | firstStep | arcE | mean |
| pitch | thigh | left | arcE | mean |
| pitch | spine | lumbar | arcE | mean |
| pitch | thigh | maxima | arcE | mean |
| pitch | thigh | minima | arcE | mean |
| pitch | thigh | right | arcE | mean |
| pitch | thigh | secondStep | arcE | mean |
| pitch | thigh | firstStep | arcE | smoothness |
| pitch | thigh | left | arcE | smoothness |
| pitch | spine | lumbar | arcE | smoothness |
| pitch | thigh | maxima | arcE | smoothness |
| pitch | thigh | minima | arcE | smoothness |
| pitch | thigh | right | arcE | smoothness |
| pitch | thigh | secondStep | arcE | smoothness |
| pitch | thigh | firstStep | arcE | smoothness2 |
| pitch | thigh | left | arcE | smoothness2 |
| pitch | spine | lumbar | arcE | smoothness2 |
| pitch | thigh | maxima | arcE | smoothness2 |
| pitch | thigh | minima | arcE | smoothness2 |
| pitch | thigh | right | arcE | smoothness2 |
| pitch | thigh | secondStep | arcE | smoothness2 |
| pitch | thigh | firstStep | arcF | AreaUnderCurve |
| pitch | thigh | left | arcF | AreaUnderCurve |
| pitch | spine | lumbar | arcF | AreaUnderCurve |
| pitch | thigh | maxima | arcF | AreaUnderCurve |
| pitch | thigh | minima | arcF | AreaUnderCurve |
| pitch | thigh | right | arcF | AreaUnderCurve |
| pitch | thigh | secondStep | arcF | AreaUnderCurve |
| pitch | thigh | firstStep | arcF | AverageUnderCurve |
| pitch | thigh | left | arcF | AverageUnderCurve |
| pitch | spine | lumbar | arcF | AverageUnderCurve |
| pitch | thigh | maxima | arcF | AverageUnderCurve |
| pitch | thigh | minima | arcF | AverageUnderCurve |
| pitch | thigh | right | arcF | AverageUnderCurve |
| pitch | thigh | secondStep | arcF | AverageUnderCurve |
| pitch | thigh | firstStep | arcF | duration |
| pitch | thigh | left | arcF | duration |
| pitch | spine | lumbar | arcF | duration |
| pitch | thigh | maxima | arcF | duration |
| pitch | thigh | minima | arcF | duration |
| pitch | thigh | right | arcF | duration |
| pitch | thigh | secondStep | arcF | duration |
| pitch | thigh | firstStep | arcF | mean |
| pitch | thigh | left | arcF | mean |
| pitch | spine | lumbar | arcF | mean |
| pitch | thigh | maxima | arcF | mean |
| pitch | thigh | minima | arcF | mean |
| pitch | thigh | right | arcF | mean |
| pitch | thigh | secondStep | arcF | mean |
| pitch | thigh | firstStep | arcF | smoothness |
| pitch | thigh | left | arcF | smoothness |
| pitch | spine | lumbar | arcF | smoothness |
| pitch | thigh | maxima | arcF | smoothness |
| pitch | thigh | minima | arcF | smoothness |
| pitch | thigh | right | arcF | smoothness |
| pitch | thigh | secondStep | arcF | smoothness |
| pitch | thigh | firstStep | arcF | smoothness2 |
| pitch | thigh | left | arcF | smoothness2 |
| pitch | spine | lumbar | arcF | smoothness2 |
| pitch | thigh | maxima | arcF | smoothness2 |
| pitch | thigh | minima | arcF | smoothness2 |
| pitch | thigh | right | arcF | smoothness2 |
| pitch | thigh | secondStep | arcF | smoothness2 |
| pitch | thigh | firstStep | arcG | AreaUnderCurve |
| pitch | thigh | left | arcG | AreaUnderCurve |
| pitch | spine | lumbar | arcG | AreaUnderCurve |
| pitch | thigh | maxima | arcG | AreaUnderCurve |
| pitch | thigh | minima | arcG | AreaUnderCurve |
| pitch | thigh | right | arcG | AreaUnderCurve |
| pitch | thigh | secondStep | arcG | AreaUnderCurve |
| pitch | thigh | firstStep | arcG | AverageUnderCurve |
| pitch | thigh | left | arcG | AverageUnderCurve |
| pitch | spine | lumbar | arcG | AverageUnderCurve |
| pitch | thigh | maxima | arcG | AverageUnderCurve |
| pitch | thigh | minima | arcG | AverageUnderCurve |
| pitch | thigh | right | arcG | AverageUnderCurve |
| pitch | thigh | secondStep | arcG | AverageUnderCurve |
| pitch | thigh | firstStep | arcG | duration |
| pitch | thigh | left | arcG | duration |
| pitch | spine | lumbar | arcG | duration |
| pitch | thigh | maxima | arcG | duration |
| pitch | thigh | minima | arcG | duration |
| pitch | thigh | right | arcG | duration |
| pitch | thigh | secondStep | arcG | duration |
| pitch | thigh | firstStep | arcG | mean |
| pitch | thigh | left | arcG | mean |
| pitch | spine | lumbar | arcG | mean |
| pitch | thigh | maxima | arcG | mean |
| pitch | thigh | minima | arcG | mean |
| pitch | thigh | right | arcG | mean |
| pitch | thigh | secondStep | arcG | mean |
| pitch | thigh | firstStep | arcG | smoothness |
| pitch | thigh | left | arcG | smoothness |
| pitch | spine | lumbar | arcG | smoothness |
| pitch | thigh | maxima | arcG | smoothness |
| pitch | thigh | minima | arcG | smoothness |
| pitch | thigh | right | arcG | smoothness |
| pitch | thigh | secondStep | arcG | smoothness |
| pitch | thigh | firstStep | arcG | smoothness2 |
| pitch | thigh | left | arcG | smoothness2 |
| pitch | spine | lumbar | arcG | smoothness2 |
| pitch | thigh | maxima | arcG | smoothness2 |
| pitch | thigh | minima | arcG | smoothness2 |
| pitch | thigh | right | arcG | smoothness2 |
| pitch | thigh | secondStep | arcG | smoothness2 |
| pitch | thigh | firstStep | arcH | AreaUnderCurve |
| pitch | thigh | left | arcH | AreaUnderCurve |
| pitch | spine | lumbar | arcH | AreaUnderCurve |
| pitch | thigh | maxima | arcH | AreaUnderCurve |
| pitch | thigh | minima | arcH | AreaUnderCurve |
| pitch | thigh | right | arcH | AreaUnderCurve |
| pitch | thigh | secondStep | arcH | AreaUnderCurve |
| pitch | thigh | firstStep | arcH | AverageUnderCurve |
| pitch | thigh | left | arcH | AverageUnderCurve |
| pitch | spine | lumbar | arcH | AverageUnderCurve |
| pitch | thigh | maxima | arcH | AverageUnderCurve |
| pitch | thigh | minima | arcH | AverageUnderCurve |
| pitch | thigh | right | arcH | AverageUnderCurve |
| pitch | thigh | secondStep | arcH | AverageUnderCurve |
| pitch | thigh | firstStep | arcH | duration |
| pitch | thigh | left | arcH | duration |
| pitch | spine | lumbar | arcH | duration |
| pitch | thigh | maxima | arcH | duration |
| pitch | thigh | minima | arcH | duration |
| pitch | thigh | right | arcH | duration |
| pitch | thigh | secondStep | arcH | duration |
| pitch | thigh | firstStep | arcH | mean |
| pitch | thigh | left | arcH | mean |
| pitch | spine | lumbar | arcH | mean |
| pitch | thigh | maxima | arcH | mean |
| pitch | thigh | minima | arcH | mean |
| pitch | thigh | right | arcH | mean |
| pitch | thigh | secondStep | arcH | mean |
| pitch | thigh | firstStep | arcH | smoothness |
| pitch | thigh | left | arcH | smoothness |
| pitch | spine | lumbar | arcH | smoothness |
| pitch | thigh | maxima | arcH | smoothness |
| pitch | thigh | minima | arcH | smoothness |
| pitch | thigh | right | arcH | smoothness |
| pitch | thigh | secondStep | arcH | smoothness |
| pitch | thigh | firstStep | arcH | smoothness2 |
| pitch | thigh | left | arcH | smoothness2 |
| pitch | spine | lumbar | arcH | smoothness2 |
| pitch | thigh | maxima | arcH | smoothness2 |
| pitch | thigh | minima | arcH | smoothness2 |
| pitch | thigh | right | arcH | smoothness2 |
| pitch | thigh | secondStep | arcH | smoothness2 |
| pitch | thigh | firstStep | arcJ | AreaUnderCurve |
| pitch | thigh | left | arcJ | AreaUnderCurve |
| pitch | spine | lumbar | arcJ | AreaUnderCurve |
| pitch | thigh | maxima | arcJ | AreaUnderCurve |
| pitch | thigh | minima | arcJ | AreaUnderCurve |
| pitch | thigh | right | arcJ | AreaUnderCurve |
| pitch | thigh | secondStep | arcJ | AreaUnderCurve |
| pitch | thigh | firstStep | arcJ | AverageUnderCurve |
| pitch | thigh | left | arcJ | AverageUnderCurve |
| pitch | spine | lumbar | arcJ | AverageUnderCurve |
| pitch | thigh | maxima | arcJ | AverageUnderCurve |
| pitch | thigh | minima | arcJ | AverageUnderCurve |
| pitch | thigh | right | arcJ | AverageUnderCurve |
| pitch | thigh | secondStep | arcJ | AverageUnderCurve |
| pitch | thigh | firstStep | arcJ | duration |
| pitch | thigh | left | arcJ | duration |
| pitch | spine | lumbar | arcJ | duration |
| pitch | thigh | maxima | arcJ | duration |
| pitch | thigh | minima | arcJ | duration |
| pitch | thigh | right | arcJ | duration |
| pitch | thigh | secondStep | arcJ | duration |
| pitch | thigh | firstStep | arcJ | mean |
| pitch | thigh | left | arcJ | mean |
| pitch | spine | lumbar | arcJ | mean |
| pitch | thigh | maxima | arcJ | mean |
| pitch | thigh | minima | arcJ | mean |
| pitch | thigh | right | arcJ | mean |
| pitch | thigh | secondStep | arcJ | mean |
| pitch | thigh | firstStep | arcJ | smoothness |
| pitch | thigh | left | arcJ | smoothness |
| pitch | spine | lumbar | arcJ | smoothness |
| pitch | thigh | maxima | arcJ | smoothness |
| pitch | thigh | minima | arcJ | smoothness |
| pitch | thigh | right | arcJ | smoothness |
| pitch | thigh | secondStep | arcJ | smoothness |
| pitch | thigh | firstStep | arcJ | smoothness2 |
| pitch | thigh | left | arcJ | smoothness2 |
| pitch | spine | lumbar | arcJ | smoothness2 |
| pitch | thigh | maxima | arcJ | smoothness2 |
| pitch | thigh | minima | arcJ | smoothness2 |
| pitch | thigh | right | arcJ | smoothness2 |
| pitch | thigh | secondStep | arcJ | smoothness2 |
| pitch | thigh | firstStep | arcK | AreaUnderCurve |
| pitch | thigh | left | arcK | AreaUnderCurve |
| pitch | spine | lumbar | arcK | AreaUnderCurve |
| pitch | thigh | maxima | arcK | AreaUnderCurve |
| pitch | thigh | minima | arcK | AreaUnderCurve |
| pitch | thigh | right | arcK | AreaUnderCurve |
| pitch | thigh | secondStep | arcK | AreaUnderCurve |
| pitch | thigh | firstStep | arcK | AverageUnderCurve |
| pitch | thigh | left | arcK | AverageUnderCurve |
| pitch | spine | lumbar | arcK | AverageUnderCurve |
| pitch | thigh | maxima | arcK | AverageUnderCurve |
| pitch | thigh | minima | arcK | AverageUnderCurve |
| pitch | thigh | right | arcK | AverageUnderCurve |
| pitch | thigh | secondStep | arcK | AverageUnderCurve |
| pitch | thigh | firstStep | arcK | duration |
| pitch | thigh | left | arcK | duration |
| pitch | spine | lumbar | arcK | duration |
| pitch | thigh | maxima | arcK | duration |
| pitch | thigh | minima | arcK | duration |
| pitch | thigh | right | arcK | duration |
| pitch | thigh | secondStep | arcK | duration |
| pitch | thigh | firstStep | arcK | mean |
| pitch | thigh | left | arcK | mean |
| pitch | spine | lumbar | arcK | mean |
| pitch | thigh | maxima | arcK | mean |
| pitch | thigh | minima | arcK | mean |
| pitch | thigh | right | arcK | mean |
| pitch | thigh | secondStep | arcK | mean |
| pitch | thigh | firstStep | arcK | smoothness |
| pitch | thigh | left | arcK | smoothness |
| pitch | spine | lumbar | arcK | smoothness |
| pitch | thigh | maxima | arcK | smoothness |
| pitch | thigh | minima | arcK | smoothness |
| pitch | thigh | right | arcK | smoothness |
| pitch | thigh | secondStep | arcK | smoothness |
| pitch | thigh | firstStep | arcK | smoothness2 |
| pitch | thigh | left | arcK | smoothness2 |
| pitch | spine | lumbar | arcK | smoothness2 |
| pitch | thigh | maxima | arcK | smoothness2 |
| pitch | thigh | minima | arcK | smoothness2 |
| pitch | thigh | right | arcK | smoothness2 |
| pitch | thigh | secondStep | arcK | smoothness2 |
| pitch | thigh | firstStep | arcL | AreaUnderCurve |
| pitch | thigh | left | arcL | AreaUnderCurve |
| pitch | spine | lumbar | arcL | AreaUnderCurve |
| pitch | thigh | maxima | arcL | AreaUnderCurve |
| pitch | thigh | minima | arcL | AreaUnderCurve |
| pitch | thigh | right | arcL | AreaUnderCurve |
| pitch | thigh | secondStep | arcL | AreaUnderCurve |
| pitch | thigh | firstStep | arcL | AverageUnderCurve |
| pitch | thigh | left | arcL | AverageUnderCurve |
| pitch | spine | lumbar | arcL | AverageUnderCurve |
| pitch | thigh | maxima | arcL | AverageUnderCurve |
| pitch | thigh | minima | arcL | AverageUnderCurve |
| pitch | thigh | right | arcL | AverageUnderCurve |
| pitch | thigh | secondStep | arcL | AverageUnderCurve |
| pitch | thigh | firstStep | arcL | duration |
| pitch | thigh | left | arcL | duration |
| pitch | spine | lumbar | arcL | duration |
| pitch | thigh | maxima | arcL | duration |
| pitch | thigh | minima | arcL | duration |
| pitch | thigh | right | arcL | duration |
| pitch | thigh | secondStep | arcL | duration |
| pitch | thigh | firstStep | arcL | mean |
| pitch | thigh | left | arcL | mean |
| pitch | spine | lumbar | arcL | mean |
| pitch | thigh | maxima | arcL | mean |
| pitch | thigh | minima | arcL | mean |
| pitch | thigh | right | arcL | mean |
| pitch | thigh | secondStep | arcL | mean |
| pitch | thigh | firstStep | arcL | smoothness |
| pitch | thigh | left | arcL | smoothness |
| pitch | spine | lumbar | arcL | smoothness |
| pitch | thigh | maxima | arcL | smoothness |
| pitch | thigh | minima | arcL | smoothness |
| pitch | thigh | right | arcL | smoothness |
| pitch | thigh | secondStep | arcL | smoothness |
| pitch | thigh | firstStep | arcL | smoothness2 |
| pitch | thigh | left | arcL | smoothness2 |
| pitch | spine | lumbar | arcL | smoothness2 |
| pitch | thigh | maxima | arcL | smoothness2 |
| pitch | thigh | minima | arcL | smoothness2 |
| pitch | thigh | right | arcL | smoothness2 |
| pitch | thigh | secondStep | arcL | smoothness2 |
| pitch | thigh | firstStep | arcM | AreaUnderCurve |
| pitch | thigh | left | arcM | AreaUnderCurve |
| pitch | spine | lumbar | arcM | AreaUnderCurve |
| pitch | thigh | maxima | arcM | AreaUnderCurve |
| pitch | thigh | minima | arcM | AreaUnderCurve |
| pitch | thigh | right | arcM | AreaUnderCurve |
| pitch | thigh | secondStep | arcM | AreaUnderCurve |
| pitch | thigh | firstStep | arcM | AverageUnderCurve |
| pitch | thigh | left | arcM | AverageUnderCurve |
| pitch | spine | lumbar | arcM | AverageUnderCurve |
| pitch | thigh | maxima | arcM | AverageUnderCurve |
| pitch | thigh | minima | arcM | AverageUnderCurve |
| pitch | thigh | right | arcM | AverageUnderCurve |
| pitch | thigh | secondStep | arcM | AverageUnderCurve |
| pitch | thigh | firstStep | arcM | duration |
| pitch | thigh | left | arcM | duration |
| pitch | spine | lumbar | arcM | duration |
| pitch | thigh | maxima | arcM | duration |
| pitch | thigh | minima | arcM | duration |
| pitch | thigh | right | arcM | duration |
| pitch | thigh | secondStep | arcM | duration |
| pitch | thigh | firstStep | arcM | mean |
| pitch | thigh | left | arcM | mean |
| pitch | spine | lumbar | arcM | mean |
| pitch | thigh | maxima | arcM | mean |
| pitch | thigh | minima | arcM | mean |
| pitch | thigh | right | arcM | mean |
| pitch | thigh | secondStep | arcM | mean |
| pitch | thigh | firstStep | arcM | smoothness |
| pitch | thigh | left | arcM | smoothness |
| pitch | spine | lumbar | arcM | smoothness |
| pitch | thigh | maxima | arcM | smoothness |
| pitch | thigh | minima | arcM | smoothness |
| pitch | thigh | right | arcM | smoothness |
| pitch | thigh | secondStep | arcM | smoothness |
| pitch | thigh | firstStep | arcM | smoothness2 |
| pitch | thigh | left | arcM | smoothness2 |
| pitch | spine | lumbar | arcM | smoothness2 |
| pitch | thigh | maxima | arcM | smoothness2 |
| pitch | thigh | minima | arcM | smoothness2 |
| pitch | thigh | right | arcM | smoothness2 |
| pitch | thigh | secondStep | arcM | smoothness2 |
| pitch | thigh | firstStep | arcN | AreaUnderCurve |
| pitch | thigh | left | arcN | AreaUnderCurve |
| pitch | spine | lumbar | arcN | AreaUnderCurve |
| pitch | thigh | maxima | arcN | AreaUnderCurve |
| pitch | thigh | minima | arcN | AreaUnderCurve |
| pitch | thigh | right | arcN | AreaUnderCurve |
| pitch | thigh | secondStep | arcN | AreaUnderCurve |
| pitch | thigh | firstStep | arcN | AverageUnderCurve |
| pitch | thigh | left | arcN | AverageUnderCurve |
| pitch | spine | lumbar | arcN | AverageUnderCurve |
| pitch | thigh | maxima | arcN | AverageUnderCurve |
| pitch | thigh | minima | arcN | AverageUnderCurve |
| pitch | thigh | right | arcN | AverageUnderCurve |
| pitch | thigh | secondStep | arcN | AverageUnderCurve |
| pitch | thigh | firstStep | arcN | duration |
| pitch | thigh | left | arcN | duration |
| pitch | spine | lumbar | arcN | duration |
| pitch | thigh | maxima | arcN | duration |
| pitch | thigh | minima | arcN | duration |
| pitch | thigh | right | arcN | duration |
| pitch | thigh | secondStep | arcN | duration |
| pitch | thigh | firstStep | arcN | mean |
| pitch | thigh | left | arcN | mean |
| pitch | spine | lumbar | arcN | mean |
| pitch | thigh | maxima | arcN | mean |
| pitch | thigh | minima | arcN | mean |
| pitch | thigh | right | arcN | mean |
| pitch | thigh | secondStep | arcN | mean |
| pitch | thigh | firstStep | arcN | smoothness |
| pitch | thigh | left | arcN | smoothness |
| pitch | spine | lumbar | arcN | smoothness |
| pitch | thigh | maxima | arcN | smoothness |
| pitch | thigh | minima | arcN | smoothness |
| pitch | thigh | right | arcN | smoothness |
| pitch | thigh | secondStep | arcN | smoothness |
| pitch | thigh | firstStep | arcN | smoothness2 |
| pitch | thigh | left | arcN | smoothness2 |
| pitch | spine | lumbar | arcN | smoothness2 |
| pitch | thigh | maxima | arcN | smoothness2 |
| pitch | thigh | minima | arcN | smoothness2 |
| pitch | thigh | right | arcN | smoothness2 |
| pitch | thigh | secondStep | arcN | smoothness2 |
| pitch | thigh | firstStep | arcP | AreaUnderCurve |
| pitch | thigh | left | arcP | AreaUnderCurve |
| pitch | spine | lumbar | arcP | AreaUnderCurve |
| pitch | thigh | maxima | arcP | AreaUnderCurve |
| pitch | thigh | minima | arcP | AreaUnderCurve |
| pitch | thigh | right | arcP | AreaUnderCurve |
| pitch | thigh | secondStep | arcP | AreaUnderCurve |
| pitch | thigh | firstStep | arcP | AverageUnderCurve |
| pitch | thigh | left | arcP | AverageUnderCurve |
| pitch | spine | lumbar | arcP | AverageUnderCurve |
| pitch | thigh | maxima | arcP | AverageUnderCurve |
| pitch | thigh | minima | arcP | AverageUnderCurve |
| pitch | thigh | right | arcP | AverageUnderCurve |
| pitch | thigh | secondStep | arcP | AverageUnderCurve |
| pitch | thigh | firstStep | arcP | duration |
| pitch | thigh | left | arcP | duration |
| pitch | spine | lumbar | arcP | duration |
| pitch | thigh | maxima | arcP | duration |
| pitch | thigh | minima | arcP | duration |
| pitch | thigh | right | arcP | duration |
| pitch | thigh | secondStep | arcP | duration |
| pitch | thigh | firstStep | arcP | mean |
| pitch | thigh | left | arcP | mean |
| pitch | spine | lumbar | arcP | mean |
| pitch | thigh | maxima | arcP | mean |
| pitch | thigh | minima | arcP | mean |
| pitch | thigh | right | arcP | mean |
| pitch | thigh | secondStep | arcP | mean |
| pitch | thigh | firstStep | arcP | smoothness |
| pitch | thigh | left | arcP | smoothness |
| pitch | spine | lumbar | arcP | smoothness |
| pitch | thigh | maxima | arcP | smoothness |
| pitch | thigh | minima | arcP | smoothness |
| pitch | thigh | right | arcP | smoothness |
| pitch | thigh | secondStep | arcP | smoothness |
| pitch | thigh | firstStep | arcP | smoothness2 |
| pitch | thigh | left | arcP | smoothness2 |
| pitch | spine | lumbar | arcP | smoothness2 |
| pitch | thigh | maxima | arcP | smoothness2 |
| pitch | thigh | minima | arcP | smoothness2 |
| pitch | thigh | right | arcP | smoothness2 |
| pitch | thigh | secondStep | arcP | smoothness2 |
| pitch | thigh | firstStep | arcQ | AreaUnderCurve |
| pitch | thigh | left | arcQ | AreaUnderCurve |
| pitch | spine | lumbar | arcQ | AreaUnderCurve |
| pitch | thigh | maxima | arcQ | AreaUnderCurve |
| pitch | thigh | minima | arcQ | AreaUnderCurve |
| pitch | thigh | right | arcQ | AreaUnderCurve |
| pitch | thigh | secondStep | arcQ | AreaUnderCurve |
| pitch | thigh | firstStep | arcQ | AverageUnderCurve |
| pitch | thigh | left | arcQ | AverageUnderCurve |
| pitch | spine | lumbar | arcQ | AverageUnderCurve |
| pitch | thigh | maxima | arcQ | AverageUnderCurve |
| pitch | thigh | minima | arcQ | AverageUnderCurve |
| pitch | thigh | right | arcQ | AverageUnderCurve |
| pitch | thigh | secondStep | arcQ | AverageUnderCurve |
| pitch | thigh | firstStep | arcQ | duration |
| pitch | thigh | left | arcQ | duration |
| pitch | spine | lumbar | arcQ | duration |
| pitch | thigh | maxima | arcQ | duration |
| pitch | thigh | minima | arcQ | duration |
| pitch | thigh | right | arcQ | duration |
| pitch | thigh | secondStep | arcQ | duration |
| pitch | thigh | firstStep | arcQ | mean |
| pitch | thigh | left | arcQ | mean |
| pitch | spine | lumbar | arcQ | mean |
| pitch | thigh | maxima | arcQ | mean |
| pitch | thigh | minima | arcQ | mean |
| pitch | thigh | right | arcQ | mean |
| pitch | thigh | secondStep | arcQ | mean |
| pitch | thigh | firstStep | arcQ | smoothness |
| pitch | thigh | left | arcQ | smoothness |
| pitch | spine | lumbar | arcQ | smoothness |
| pitch | thigh | maxima | arcQ | smoothness |
| pitch | thigh | minima | arcQ | smoothness |
| pitch | thigh | right | arcQ | smoothness |
| pitch | thigh | secondStep | arcQ | smoothness |
| pitch | thigh | firstStep | arcQ | smoothness2 |
| pitch | thigh | left | arcQ | smoothness2 |
| pitch | spine | lumbar | arcQ | smoothness2 |
| pitch | thigh | maxima | arcQ | smoothness2 |
| pitch | thigh | minima | arcQ | smoothness2 |
| pitch | thigh | right | arcQ | smoothness2 |
| pitch | thigh | secondStep | arcQ | smoothness2 |
| pitch | thigh | firstStep | arcR | AreaUnderCurve |
| pitch | thigh | left | arcR | AreaUnderCurve |
| pitch | spine | lumbar | arcR | AreaUnderCurve |
| pitch | thigh | maxima | arcR | AreaUnderCurve |
| pitch | thigh | minima | arcR | AreaUnderCurve |
| pitch | thigh | right | arcR | AreaUnderCurve |
| pitch | thigh | secondStep | arcR | AreaUnderCurve |
| pitch | thigh | firstStep | arcR | AverageUnderCurve |
| pitch | thigh | left | arcR | AverageUnderCurve |
| pitch | spine | lumbar | arcR | AverageUnderCurve |
| pitch | thigh | maxima | arcR | AverageUnderCurve |
| pitch | thigh | minima | arcR | AverageUnderCurve |
| pitch | thigh | right | arcR | AverageUnderCurve |
| pitch | thigh | secondStep | arcR | AverageUnderCurve |
| pitch | thigh | firstStep | arcR | duration |
| pitch | thigh | left | arcR | duration |
| pitch | spine | lumbar | arcR | duration |
| pitch | thigh | maxima | arcR | duration |
| pitch | thigh | minima | arcR | duration |
| pitch | thigh | right | arcR | duration |
| pitch | thigh | secondStep | arcR | duration |
| pitch | thigh | firstStep | arcR | mean |
| pitch | thigh | left | arcR | mean |
| pitch | spine | lumbar | arcR | mean |
| pitch | thigh | maxima | arcR | mean |
| pitch | thigh | minima | arcR | mean |
| pitch | thigh | right | arcR | mean |
| pitch | thigh | secondStep | arcR | mean |
| pitch | thigh | firstStep | arcR | smoothness |
| pitch | thigh | left | arcR | smoothness |
| pitch | spine | lumbar | arcR | smoothness |
| pitch | thigh | maxima | arcR | smoothness |
| pitch | thigh | minima | arcR | smoothness |
| pitch | thigh | right | arcR | smoothness |
| pitch | thigh | secondStep | arcR | smoothness |
| pitch | thigh | firstStep | arcR | smoothness2 |
| pitch | thigh | left | arcR | smoothness2 |
| pitch | spine | lumbar | arcR | smoothness2 |
| pitch | thigh | maxima | arcR | smoothness2 |
| pitch | thigh | minima | arcR | smoothness2 |
| pitch | thigh | right | arcR | smoothness2 |
| pitch | thigh | secondStep | arcR | smoothness2 |
| pitch | thigh | firstStep | sit2stand | peakAbs |
| pitch | thigh | left | sit2stand | peakAbs |
| pitch | thigh | maxima | sit2stand | peakAbs |
| pitch | thigh | minima | sit2stand | peakAbs |
| pitch | thigh | right | sit2stand | peakAbs |
| pitch | thigh | secondStep | sit2stand | peakAbs |
| pitch | thigh | firstStep | sit2stand | peakValue |
| pitch | thigh | left | sit2stand | peakValue |
| pitch | thigh | maxima | sit2stand | peakValue |
| pitch | thigh | minima | sit2stand | peakValue |
| pitch | thigh | right | sit2stand | peakValue |
| pitch | thigh | secondStep | sit2stand | peakValue |
| pitch | spine | lumbar | sit2stand | spine2outward15pcDelay |
| pitch | spine | lumbar | sit2stand | spine2thighDelay |
| pitch | spine | lumbar | sit2stand | weissDuration |
| pitch | spine | lumbar | sit2stand1 | peakAbs |
| pitch | spine | lumbar | sit2stand1 | peakValue |
| pitch | spine | lumbar | sit2stand2 | peakAbs |
| pitch | spine | lumbar | sit2stand2 | peakValue |
| pitch | thigh | firstStep | stand2sit | peakAbs |
| pitch | thigh | left | stand2sit | peakAbs |
| pitch | thigh | maxima | stand2sit | peakAbs |
| pitch | thigh | minima | stand2sit | peakAbs |
| pitch | thigh | right | stand2sit | peakAbs |
| pitch | thigh | secondStep | stand2sit | peakAbs |
| pitch | thigh | firstStep | stand2sit | peakValue |
| pitch | thigh | left | stand2sit | peakValue |
| pitch | thigh | maxima | stand2sit | peakValue |
| pitch | thigh | minima | stand2sit | peakValue |
| pitch | thigh | right | stand2sit | peakValue |
| pitch | thigh | secondStep | stand2sit | peakValue |
| pitch | spine | lumbar | stand2sit | weissDuration |
| pitch | spine | lumbar | stand2sit1 | peakAbs |
| pitch | spine | lumbar | stand2sit1 | peakValue |
| pitch | spine | lumbar | stand2sit2 | peakAbs |
| pitch | spine | lumbar | stand2sit2 | peakValue |
| roll | spine | lumbar | arcA | AreaUnderCurve |
| roll | spine | lumbar | arcA | AverageUnderCurve |
| roll | spine | lumbar | arcA | duration |
| roll | spine | lumbar | arcA | mean |
| roll | spine | lumbar | arcA | smoothness |
| roll | spine | lumbar | arcA | smoothness2 |
| roll | spine | lumbar | arcB | AreaUnderCurve |
| roll | spine | lumbar | arcB | AverageUnderCurve |
| roll | spine | lumbar | arcB | duration |
| roll | spine | lumbar | arcB | mean |
| roll | spine | lumbar | arcB | smoothness |
| roll | spine | lumbar | arcB | smoothness2 |
| roll | spine | lumbar | arcC | AreaUnderCurve |
| roll | spine | lumbar | arcC | AverageUnderCurve |
| roll | spine | lumbar | arcC | duration |
| roll | spine | lumbar | arcC | mean |
| roll | spine | lumbar | arcC | smoothness |
| roll | spine | lumbar | arcC | smoothness2 |
| roll | spine | lumbar | arcD | AreaUnderCurve |
| roll | spine | lumbar | arcD | AverageUnderCurve |
| roll | spine | lumbar | arcD | duration |
| roll | spine | lumbar | arcD | mean |
| roll | spine | lumbar | arcD | smoothness |
| roll | spine | lumbar | arcD | smoothness2 |
| roll | spine | lumbar | arcE | AreaUnderCurve |
| roll | spine | lumbar | arcE | AverageUnderCurve |
| roll | spine | lumbar | arcE | duration |
| roll | spine | lumbar | arcE | mean |
| roll | spine | lumbar | arcE | smoothness |
| roll | spine | lumbar | arcE | smoothness2 |
| roll | spine | lumbar | arcF | AreaUnderCurve |
| roll | spine | lumbar | arcF | AverageUnderCurve |
| roll | spine | lumbar | arcF | duration |
| roll | spine | lumbar | arcF | mean |
| roll | spine | lumbar | arcF | smoothness |
| roll | spine | lumbar | arcF | smoothness2 |
| roll | spine | lumbar | arcG | AreaUnderCurve |
| roll | spine | lumbar | arcG | AverageUnderCurve |
| roll | spine | lumbar | arcG | duration |
| roll | spine | lumbar | arcG | mean |
| roll | spine | lumbar | arcG | smoothness |
| roll | spine | lumbar | arcG | smoothness2 |
| roll | spine | lumbar | arcH | AreaUnderCurve |
| roll | spine | lumbar | arcH | AverageUnderCurve |
| roll | spine | lumbar | arcH | duration |
| roll | spine | lumbar | arcH | mean |
| roll | spine | lumbar | arcH | smoothness |
| roll | spine | lumbar | arcH | smoothness2 |
| roll | spine | lumbar | arcJ | AreaUnderCurve |
| roll | spine | lumbar | arcJ | AverageUnderCurve |
| roll | spine | lumbar | arcJ | duration |
| roll | spine | lumbar | arcJ | mean |
| roll | spine | lumbar | arcJ | smoothness |
| roll | spine | lumbar | arcJ | smoothness2 |
| roll | spine | lumbar | arcK | AreaUnderCurve |
| roll | spine | lumbar | arcK | AverageUnderCurve |
| roll | spine | lumbar | arcK | duration |
| roll | spine | lumbar | arcK | mean |
| roll | spine | lumbar | arcK | smoothness |
| roll | spine | lumbar | arcK | smoothness2 |
| roll | spine | lumbar | arcL | AreaUnderCurve |
| roll | spine | lumbar | arcL | AverageUnderCurve |
| roll | spine | lumbar | arcL | duration |
| roll | spine | lumbar | arcL | mean |
| roll | spine | lumbar | arcL | smoothness |
| roll | spine | lumbar | arcL | smoothness2 |
| roll | spine | lumbar | arcM | AreaUnderCurve |
| roll | spine | lumbar | arcM | AverageUnderCurve |
| roll | spine | lumbar | arcM | duration |
| roll | spine | lumbar | arcM | mean |
| roll | spine | lumbar | arcM | smoothness |
| roll | spine | lumbar | arcM | smoothness2 |
| roll | spine | lumbar | arcN | AreaUnderCurve |
| roll | spine | lumbar | arcN | AverageUnderCurve |
| roll | spine | lumbar | arcN | duration |
| roll | spine | lumbar | arcN | mean |
| roll | spine | lumbar | arcN | smoothness |
| roll | spine | lumbar | arcN | smoothness2 |
| roll | spine | lumbar | arcP | AreaUnderCurve |
| roll | spine | lumbar | arcP | AverageUnderCurve |
| roll | spine | lumbar | arcP | duration |
| roll | spine | lumbar | arcP | mean |
| roll | spine | lumbar | arcP | smoothness |
| roll | spine | lumbar | arcP | smoothness2 |
| roll | spine | lumbar | arcQ | AreaUnderCurve |
| roll | spine | lumbar | arcQ | AverageUnderCurve |
| roll | spine | lumbar | arcQ | duration |
| roll | spine | lumbar | arcQ | mean |
| roll | spine | lumbar | arcQ | smoothness |
| roll | spine | lumbar | arcQ | smoothness2 |
| roll | spine | lumbar | arcR | AreaUnderCurve |
| roll | spine | lumbar | arcR | AverageUnderCurve |
| roll | spine | lumbar | arcR | duration |
| roll | spine | lumbar | arcR | mean |
| roll | spine | lumbar | arcR | smoothness |
| roll | spine | lumbar | arcR | smoothness2 |
| roll | spine | lumbar | sit2stand | spine2outward15pcDelay |
| roll | spine | lumbar | sit2stand | spine2thighDelay |
| roll | spine | lumbar | sit2stand | weissDuration |
| roll | spine | lumbar | sit2stand1 | baseline |
| roll | spine | lumbar | sit2stand1 | peakAbs |
| roll | spine | lumbar | sit2stand2 | baseline |
| roll | spine | lumbar | sit2stand2 | peakAbs |
| roll | spine | lumbar | stand2sit | weissDuration |
| roll | spine | lumbar | stand2sit1 | baseline |
| roll | spine | lumbar | stand2sit1 | peakAbs |
| roll | spine | lumbar | stand2sit2 | baseline |
| roll | spine | lumbar | stand2sit2 | peakAbs |
